# Distinct regulation of alternative polyadenylation and gene expression by nuclear poly(A) polymerases

**DOI:** 10.1093/nar/gkx560

**Published:** 2017-06-27

**Authors:** Weimin Li, Wencheng Li, Rakesh S. Laishram, Mainul Hoque, Zhe Ji, Bin Tian, Richard A. Anderson

**Affiliations:** 1 University of Wisconsin-Madison, School of Medicine and Public Health, Madison, WI 53706, USA; 2 Washington State University, Elson S. Floyd College of Medicine, Department of Biomedical Sciences, Spokane, WA 99202, USA; 3 Rutgers New Jersey Medical School, Department of Microbiology, Biochemistry and Molecular Genetics, Newark, NJ 07103, USA

## Abstract

Polyadenylation of nascent RNA by poly(A) polymerase (PAP) is important for 3′ end maturation of almost all eukaryotic mRNAs. Most mammalian genes harbor multiple polyadenylation sites (PASs), leading to expression of alternative polyadenylation (APA) isoforms with distinct functions. How poly(A) polymerases may regulate PAS usage and hence gene expression is poorly understood. Here, we show that the nuclear canonical (PAPα and PAPγ) and non-canonical (Star-PAP) PAPs play diverse roles in PAS selection and gene expression. Deficiencies in the PAPs resulted in perturbations of gene expression, with Star-PAP impacting lowly expressed mRNAs and long-noncoding RNAs to the greatest extent. Importantly, different PASs of a gene are distinctly regulated by different PAPs, leading to widespread relative expression changes of APA isoforms. The location and surrounding sequence motifs of a PAS appear to differentiate its regulation by the PAPs. We show Star-PAP-specific PAS usage regulates the expression of the eukaryotic translation initiation factor *EIF4A1*, the tumor suppressor gene *PTEN* and the long non-coding RNA *NEAT1*. The Star-PAP-mediated APA of *PTEN* is essential for DNA damage-induced increase of PTEN protein levels. Together, our results reveal a PAS-guided and PAP-mediated paradigm for gene expression in response to cellular signaling cues.

## INTRODUCTION

Most eukaryotic mRNAs employ cleavage and polyadenylation (CPA) for 3′ end maturation (1,2). After cleavage of the RNA polymerase II-bound nascent RNA, the RNA is polyadenylated to ∼250 nt in mammalian cells and ∼50 nt in yeast (3). The poly(A) tail is important for mRNA metabolism at multiple levels, including nuclear export, mRNA stability and translation (4). Dynamic regulation of the poly(A) tail length through deadenylation and cytoplasmic polyadenylation plays an important role in early development and neuronal functions (3,4).

The machinery responsible for CPA includes several subcomplexes (5), such as the Cleavage and Polyadenylation Specificity Factor (CPSF), comprising CPSF-160, CPSF-100, CPSF-73, CPSF-30, Fip1 and WDR33; the Cleavage Stimulation Factor (CstF), comprising CstF-50, CstF-64/CstF-64τ and CstF-77; the Cleavage Factor (CF) I, comprising CFI-25, CFI-59 and CFI-68; CF II, comprising Pcf11 and Clp1; and a number of single proteins, such as symplekin, nuclear poly(A) binding protein (PABPN1) and poly(A) polymerase (PAP).

Three canonical nuclear PAPs have been identified in mammalian cells, including PAPα and PAPγ/neo-PAP, both of which are ubiquitously expressed PAPs (6,7), and PAPβ, whose expression appears to be restricted to testis (8). Star-PAP, also known as Tut1, is a nuclear, non-canonical PAP that also has terminal uridylyltransferase activity. Star-PAP can be regulated by the lipid messenger phosphatidylinositol-4,5-bisphosphate (PI4,5P_2_) and its associated protein kinases (9–13). Previous studies have shown that Star-PAP directly binds target pre-mRNAs upstream of the PAS and recruits CPSF for 3′ end cleavage (10,12). In contrast, the canonical PAPα and PAPγ displayed low affinity for RNA (14), and their interactions with substrate RNA require the CPA machinery (15). Additionally, Star-PAP and PAPα appear to be mutually exclusive in mRNA processing complexes, and certain Star-PAP associated kinases were not detected in the complex containing PAPα (13).

Most protein-coding genes in eukaryotes have multiple PASs to generate alternative polyadenylation (APA) mRNA isoforms (16–20). APA occurring in 3′ terminal exons typically changes the length of 3′ untranslated region (3′UTR), whereas APA in upstream introns or exons leads to isoforms with different coding sequences (CDS) and 3′UTRs. Consequently, APA isoforms have different post-transcriptional properties and/or protein-coding potentials. APA has been shown to be dynamically regulated across tissues and under different cellular conditions (21,22). For example, it was reported that APA caused global shortening of 3′UTRs in proliferating cells compared to quiescent cells (23,24). Conversely, progressive lengthening of 3′UTRs was observed during cell differentiation and early development (25,26).

A number of mechanisms have been found to regulate APA (19,27), including core CPA factors, various RNA-binding proteins (RBPs) and splicing factors. Most of the factors are believed to regulate the cleavage step of CPA. For example, knockdown (KD) of CFI-25 or CFI-68 caused global 3′UTR shortening whereas inhibition of Pcf11, Fip1 or CstF-64+CstF-64τ led to global 3′UTR lengthening (28,29). However, factors involved in the polyadenylation step have also been implicated in APA. First, PABPN1, generally believed to be important for the polyadenylation step only, has been shown to regulate PAS choice (30,31). Second, inhibition of PAPα by U1 snRNP has long been shown to regulate transcript expression (32). Consistently, functional inhibition of U1 snRNP caused activation of intronic polyadenylation (28,33), a mechanism with implications in neuronal activation (34,35) and cellular response to UV-induced DNA damage (36).

Star-PAP was previously shown to control CPA of select genes with a single PAS in response to oxidative stress and DNA damage signals (10,12,13). Here, using a high throughput 3′ end sequencing, we show genome-wide APA regulation landscapes by Star-PAP, PAPα and PAPγ. We have validated APA of several key genes regulated by Star-PAP, including the eukaryotic translation initiation factor *EIF4A1*, the tumor suppressor phosphatase and tensin homolog deleted on chromosome 10 (*PTEN*), and the long non-coding RNA *NEAT1*. Our data indicate that individual PAPs selectively target specific PASs to regulate their usage, providing a new paradigm for the modulation of gene expression.

## MATERIALS AND METHODS

### Cell culture, transfection and treatments

Human embryonic kidney (HEK) 293 cells were obtained from American Type Culture Collection. The cell culture conditions and the transfections with the plasmids containing the wild-type (wt) or polymerase dead (pd) Star-PAP with RNAi-insensitive silent mutations (sm), wt PAPα, pLightSwitch_*PTEN* 3′UTR, pLightSwitch_*GAPDH* 3′UTR, pLightSwitch_*EIF4A1* 3′UTR-wt/mut and pLightSwitch_*NEAT1* RNA-wt/mut without the SV40 poly(A) sequence originally contained in the empty pLightSwitch_3′UTR vector (SwitchGear Genomics), as well as the siRNAs (listed in the Supplementary Data) were performed as previously described (12). The luciferase activity was measured 48 h post-transfection using the LightSwitch Assay Reagents and the BioTek Synergy 2 microplate reader (BellBrook Labs) according to the manufacturers’ instructions. The *PTEN* 3′UTR sequence inserted into the luciferase expressing vector contained the full length 3′UTR and 132 nt downstream of the most distal PAS. The *GAPDH* 3′UTR sequence inserted into the luciferase expressing vector contained the full length 3′UTR and extended 130 nt downstream of the distal PAS. The distal PAS-flanking sequences of the *EIF4A1* and *NEAT1* RNAs were 120 and 100 nt upstream and downstream, respectively, of the distal PAS site (Supplementary Figure S6). Whenever required, cells were treated with 50 μM of etoposide or the vehicle control, dimethyl sulfoxide (DMSO), for 6 h or the indicated lengths of time and harvested for analysis.

### Validation assays

Gene expression analysis using quantitative real-time RT-PCR (qRT-PCR) was carried out as described previously (12). The primers used are listed in the Supplementary Data. GAPDH expression was used as an internal control for normalization. Star-PAP regulation of mRNA isoform expression by using select PASs was evaluated using qRT-PCR analysis over the expression of the reference sequence within the same gene but was not affected by the siRNA KD of the PAPs. The siRNAs used in the validation experiments were the same as those used for global analyses. At least two individual siRNAs were tested for knockdown specificities (Supplementary Figure S1A). Immunoblotting (IB) was carried out as described (12). RNA immunoprecipitation and quantitative Real-Time RT-PCR analysis for the PAP-associated RNA expression were carried out as described previously (12).

### 3′READS

Deep sequencing of mRNA was based on the 3′ region extraction and deep sequencing (3′READS) method (37). Briefly, total RNA was subjected to one round of poly(A) selection using the Poly(A)Purist™ MAG kit (Ambion), followed by fragmentation using the RNA fragmentation kit (Ambion). Poly(A)-containing RNA fragments were isolated using the MyOne streptavidin C1 beads (Invitrogen) coated with a 5′ biotinylated chimeric U_5_T_45_ oligo (Sigma) at room temperature for 1 hr in the binding buffer (10 mM Tris–HCl pH 7.5, 150 mM NaCl, 1 mM ethylenediaminetetraacetic acid (EDTA)), followed by washing with the washing buffer (10 mM Tris–HCl pH7.5, 1 mM NaCl, 1 mM EDTA, 10% Formamide). RNA bound to the beads was digested with RNase H (5U in 50 μl reaction volume) at 37°C for 1 h, which removed the poly(A) sequence and eluted RNA from the beads. Eluted RNA fragments were purified by phenol:chloroform extraction and ethanol precipitation, followed by phosphorylation of the 5′ end with T4 kinase (NEB). Phosphorylated RNA was then purified by the RNeasy kit (Qiagen) and was sequentially ligated to a 5′-adenylated 3′ adapter (5′-rApp/NNNNGATCGTCGGACTGTAGAACTCTGAAC/3ddC) with the truncated T4 RNA ligase II (Bioo Scientific) and to a 5′ adapter (5′-GUUCAGAGUUCUACAGUCCGACGAUC) by T4 RNA ligase I (NEB). The resultant RNA was reverse-transcribed to cDNA with Superscript III (Invitrogen), followed by 12 cycles of polymerase chain reaction (PCR) amplification with Phusion high fidelity polymerase (NEB). cDNA libraries were sequenced on an Illumina Hiseq 2000 sequencer (1 × 100 nt).

### Identification of PASs

3′READS reads were mapped to the human genome (hg19) using bowtie2 (local mode). Uniquely mapped reads (with MAPQ score > 10) that had at least two additional 5′ Ts not aligned to genome (presumably derived from the poly(A) tail) were named PAS supporting (PASS) reads and were used to calculate PAS isoform expression. PASs located within 24 nt were iteratively clustered as previously described (38), and the site with the greatest number of PASS reads in a cluster was identified as the representative PAS for the cluster. We required a PAS to be supported by at least two PASS reads and the relative abundance of transcript using the PAS to be >5% in a gene in at least one sample.

### Gene and PAS-based transcript expression analyses

The gene or PAS expression level was calculated as the total PASS reads assigned for a gene or one PAS, and was measured by the RPM value (number of reads per million uniquely mapped PASS reads in a sample). mRNAs and long non-coding RNAs (lncRNAs) annotations were obtained from the Refseq database. The expression change of a gene or PAS between two samples (e.g. KD and siCtrl) was calculated by the log 2 based ratio of their RPM values. For relative PAS-based transcript expression analysis, the Significance Analysis of Alternative Polyadenylation (SAAP) method (Li *et al.*, (28)) was used to examine whether the relative expression of two PAS isoforms (e.g. two PASs in 3′UTR) or PAS groups (e.g. PASs in upstream regions versus PASs in 3′-most exons) is significantly different between two comparing samples. For 3′UTR APA, relative expression difference (RED) was calculated as log2Ratio of read number of distal PAS isoform to that of proximal PAS isoform.

### Star-PAP-binding motif score

Star-PAP binding Position Frequency Matrix (PFM) was downloaded from cisBP-RNA website (Ray *et al.*, (39)). The PFM of Star-PAP was a seven by four position matrix. We used this PFM to scan the −100 to +100 region of each PAS identified from our 3′READS data. The Star-PAP-binding motif score was calculated as follows: for each position of a given sequence, a seven nucleotide (7-mer) region starting at the position was examined. The score for the 7-mer was calculated as $\sum\nolimits_{p = 1}^7 {(fp,n*Ip)}$, Where *fp, n* is the relative frequency of the nucleotide *n* at position *p* as defined in the PFM, and *I_p_* is the information content of position *p*. $Ip = 2 - ( { - \sum\nolimits_{n = 1}^4 {({f_n}lo{g_2}{f_n})} } )$, where *f_n_* (*n* = 1 to 4) is relative frequency of each of the four nucleotides. Scanning was carried out by a PERL script, and results were plotted using the program R with lines smoothed by the LOWESS function (smoother span *f* = 0.05).

### Other bioinformatics analyses

Gene Ontology (GO) analysis was based on the Fisher's exact test. GO annotation of genes was obtained from the NCBI Gene database. To identify sequence motifs around PASs, we separated PASs into different groups based on their isoform expression change after PAP KDs. Frequencies of k-mers (*k* = 4) in different regions (e.g. −100 to −41 nt upstream of the PAS) were counted and compared using the Fisher's exact test.

### RNA immunoprecipitation and quantitative Real-Time RT-PCR

As described previously (12).

## RESULTS

### Nuclear PAPs regulate transcript abundance genome-wide

**Figure 1.**
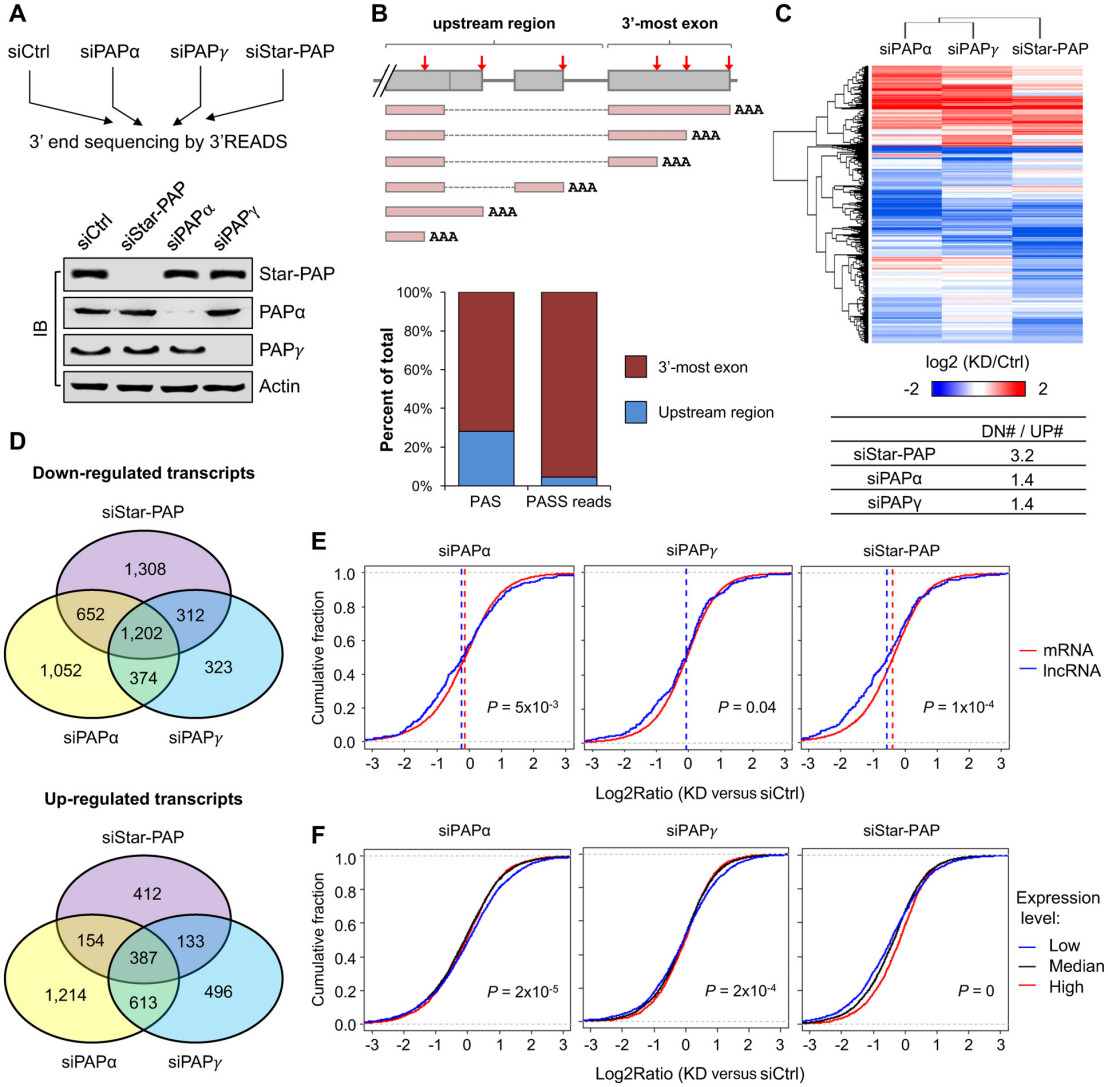
Genome-wide regulation of gene expression by PAPs. (**A**) Top, experimental design. HEK 293 cells were used. Bottom, the knockdown efficiencies of the individual PAPs were examined by Immunoblot (IB). (**B**) Top, schematic of PASs in a gene. Bottom, distribution of identified PASs and PASS reads from 3′READS. PASS reads were those with at least two non-genomic ‘A’s at the end. (**C**) Change of transcript expression level after KD of Star-PAP, PAPα or PAPγ. Each transcript was based on one PAS. Those with fold change >2 and total read counts >50 were plotted using the color scale shown at bottom. Samples were clustered using hierarchical clustering with Pearson Correlation coefficient as metric. The ratio of number of downregulated transcript to that of upregulated ones in each KD was shown in a table below the heatmap. (**D**) Venn diagrams comparing transcripts similarly or differently downregulated (top) or upregulated (bottom) upon each PAP KD. (**E**) Expression profiles of mRNAs and lncRNAs in each KD sample. (**F**) Expression levels of the different genes affected by PAP KD. Low, median and high expression genes were those <25th, 25th–75th, >75th percentiles, respectively. *P*-value was based on K-S test comparing <25th with >75th groups.

PAPα, PAPγ and Star-PAP are three currently known, ubiquitously expressed nuclear PAPs (6,13). We asked whether these PAPs regulate the expression of transcripts using different PASs. To this end, we knocked down the expression of these PAPs individually as well as knocked down both PAPα and PAPγ at the same time in HEK293 cells using siRNAs (Supplementary Figure S1), and subjected total cellular RNA to the 3′ end-based RNA sequencing method, 3′ region extraction and deep sequencing (3′READS) (37). The efficiencies of the KDs were confirmed by Western Blot analysis (Figure 1A). Each PAP KD was specific and did not appear to change the expression of the other PAPs (Figure 1A and Supplementary Figure S1). We obtained 4–25 million PAS-supporting (PASS) reads in each of the experimental conditions (Supplementary Table S1), corresponding to 57 802 unique PASs in 15 702 RefSeq-annotated genes. Most reads (96%) were mapped to RefSeq-defined 3′-most exons and 3′ extended regions (Figure 1B, upper panel), corresponding to 72% of all PASs. In contrast, sites located upstream of the 3′-most exon accounted for 28% of all PASs and 4% of all PASS reads (Figure 1B, lower panel), consistent with their low expression in general compared to 3′-most exon PASs (28).

We next examined how transcripts with specific PASs were regulated in each KD sample based on their PAS. As such, multiple transcripts can belong to the same gene due to APA. Using total read number >50 and fold change >2 as the cutoff for regulation, 8,664 PAS-specific transcripts, corresponding to 6481 genes were selected (Figure 1C). Cluster analysis indicated that transcript expression profiles of siPAPα and siPAPγ samples appeared similar compared to that of siStar-PAP (Figure 1C). Overall, more transcripts were downregulated than upregulated in expression across all of the KD samples (Figure 1C), indicating that all these PAPs are important for proper mRNA expression. This bias was most conspicuous with the siStar-PAP sample, which had 3.2-fold more downregulated transcripts than upregulated ones. In contrast, the bias was 1.4-fold for the siPAPα or siPAPγ samples (Figure 1C).

We then specifically compared regulated transcripts between three KD samples (Figure 1D). About 25–35% of the regulated transcripts, either upregulated or downregulated, in each KD sample were similarly regulated in two other KD samples as well (Figure 1D) and another 27–40% of the transcripts were similarly regulated in at least another KD sample, indicating overlapping functions between the PAPs. However, about 21–44% of the regulated transcripts in each sample were unique to each individual PAP KD (Figure 1D), indicating specificity of different PAPs. Interestingly, more transcripts showed specific downregulation in the siStar-PAP (44.0%) and siPAPα (38.4%) samples than in the siPAPγ (24.2%) sample, indicative of unique features of Star-PAP and PAPα.

Next, we examined the effect of gene expression in different PAP KDs. The expression of a gene was defined as the sum of expression (RPM values) of all the APA isoforms in a gene. As expected, we found that genes were more downregulated by siStar-PAP than by siPAPα or siPAPγ (Figure 1E, compare the median values of the three KD samples). Interestingly, the long non-coding RNA (lncRNA) genes were downregulated to a greater extent than mRNA genes by siStar-PAP (Figure 1E), which was less obvious in siPAPα and siPAPγ samples. We found consistent trends of regulation in different batches of 3′READS experiments for siStar-PAP and knockdown of both PAPα and PAPγ at the same time (Supplementary Table S1 and Figure S2A). Further analysis also indicated that genes expressed at low levels were more likely to be downregulated by siStar-PAP, but less so by siPAPα or siPAPγ (Figure 1F and Supplementary Figure S2B). Therefore, it appears that lowly expressed genes and lncRNAs are particularly reliant on Star-PAP for expression.

Analysis of GO information revealed that each of the nuclear PAPs controlled select sets of genes involved in distinct biological functions (Supplementary Table S2). For instance, genes downregulated by siPAPα tended to function in post-embryonic development; those downregulated by siPAPγ appeared to be involved in cell-type specific apoptotic process; and genes downregulated by siStar-PAP tended to participate in regulation of chromosome organization. By contrast, genes downregulated by all three KDs tended to have functions in neuron differentiation, extracellular matrix organization, response to organic cyclic compound, etc. (Supplementary Table S2). Taken together, our genome-wide profiling of PAS-specific transcripts and genes indicates that a large number of genes depend on PAPs for expression, and different PAPs influence the abundance of transcripts using different PASs to variable degrees.

### Selective PAS usage modulated by PAPs

**Figure 2.**
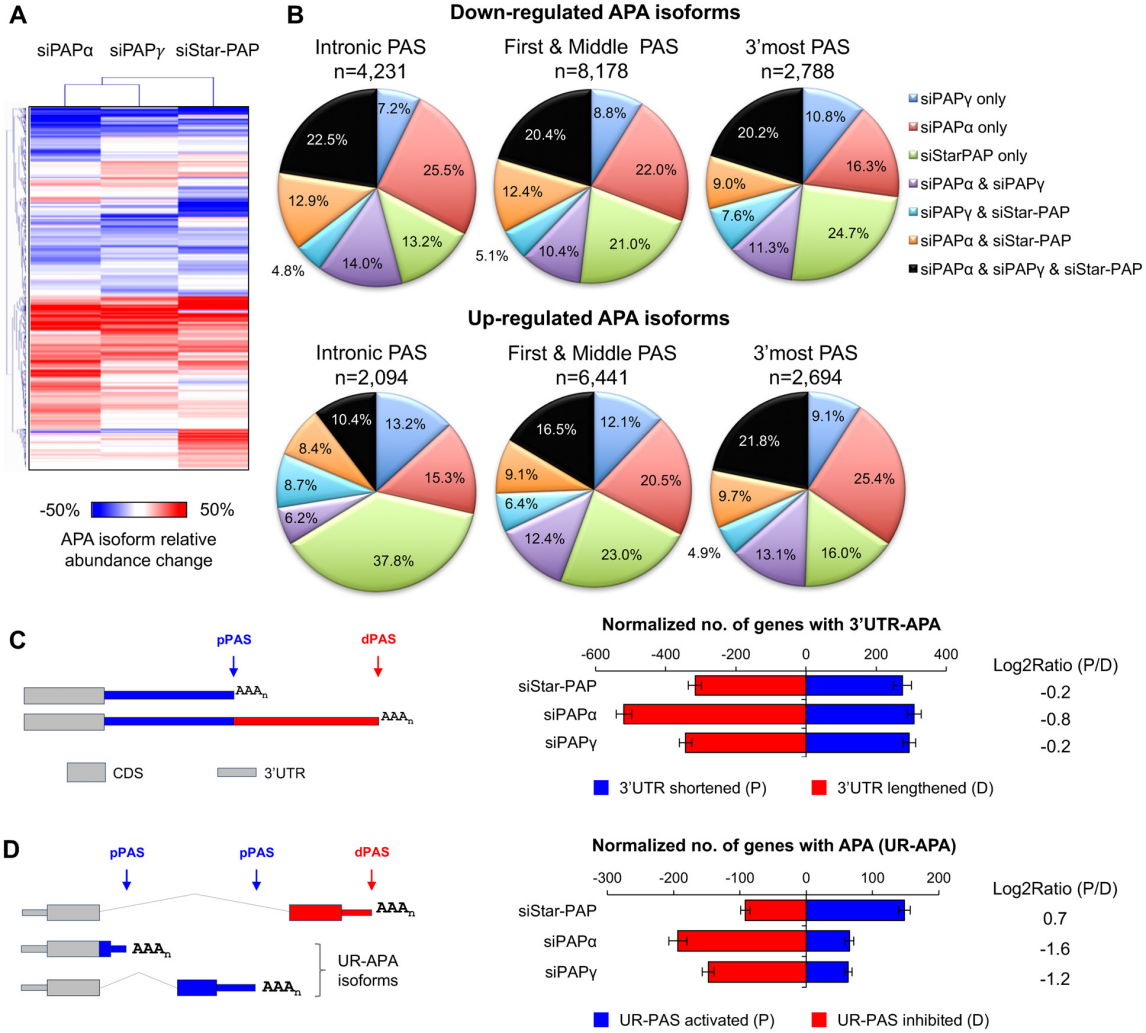
Widespread APA events regulated by PAPs. (**A**) Heatmap showing APA changes in three KD samples. Only those with significant changes (FDR < 0.05, Significance Analysis of Alternative Polyadenylation; SAAP) were selected and shown in the heatmap using the color scale shown below the plot. Samples were clustered using the hierarchical clustering with Pearson correlation coefficient as metric. (**B**) Pie charts summarizing commonly or uniquely regulated APA events. (**C**) Left, schematic of 3′UTR-APA. Right, regulation of 3′UTR-APA as analyzed by Global Analysis of Alternative Polyadenylation (GAAP). The log2Ratio of the number of genes with 3′UTR shortened (P for proximal) to the number of genes with 3′UTR lengthened (D for distal) was shown. (**D**) Left, schematic of UR-APA. Right, regulation of UR-APA as analyzed by GAAP. Right, the log2Ratio of the number of genes with UR-PAS usage activated to the number of genes with UR-PAS usage inhibited was shown.

The overlapping yet different coverage of PAS-specific transcripts by the PAPs (Figure 1C and D) suggests that the individual PASs are selectively processed by different PAPs. To address this hypothesis more specifically, we examined relative expression of APA isoforms using different PASs in the same gene. Using the Significance Analysis of Alternative Polyadenylation (SAAP) method (28) (see ‘Materials and Methods’ for detail), we identified 8037 genes with significant APA regulation in the KD samples (Figure 2A and Supplementary Figure S3A). Similar to transcript-based analysis result, a large fraction of regulated APA events were common to the three KD samples (Figure 2B), indicating overlapping functions of the three nuclear PAPs. Uniquely regulated APA events were also frequent, especially in siPAPα and siStar-PAP samples (Figure 2B). Interestingly, isoforms using 3′-most PASs tended to be downregulated upon Star-PAP KD whereas those using intronic PASs tended to be upregulated under the same condition (Figure 2B). In contrast, loss of PAPα showed an opposite trend (Figure 2B). The PAS location bias is modest in the PAPγ KD, which is globally more similar to PAPα KD than to Star-PAP KD (Figure 2A and B; Supplementary Figure S3A).

We next asked whether PAP KDs display global trends of APA as seen with several other CPA factors (28). Using the Global Analysis of Alternative Polyadenylation method to assess the general trend of APA by taking the variation of sequencing depth into account (see ‘Materials and Methods’ section), we found that there was only mild global lengthening of 3′UTRs in the KD samples (Figure 2C and Supplementary Figure S3B), with the log2Ratio of the number of genes with 3′UTR shortening to 3′UTR lengthening being −0.2 to −0.8 for the KD samples. siPAPα showed more 3′UTR lengthening than other KDs. We then examined APA isoforms using upstream region PASs (UR-PAS) (Figure 2D, left panel). Interestingly, siPAPα and siPAPγ elicited general suppression of the usage of these PASs, whereas a mild activation of UR-PAS usage was observed with the siStar-PAP sample (Figure 2D, right panel and Supplementary Figure S3C). These results again indicate that each PAP regulates select APA isoforms with location preferences. Consistent with this, GO analysis showed that genes with regulated APA after each PAP KD were enriched for distinct GO terms (Supplementary Table S3). However, most significant GO terms were associated with genes whose APA was affected by all three PAPs, such as mRNA metabolic process, RNA processing, protein folding, etc. (Supplementary Table S3), suggesting their APA expression is highly sensitive to PAP activities in general.

### Sequence-dependent regulation of APA isoforms by Star-PAP

**Figure 3.**
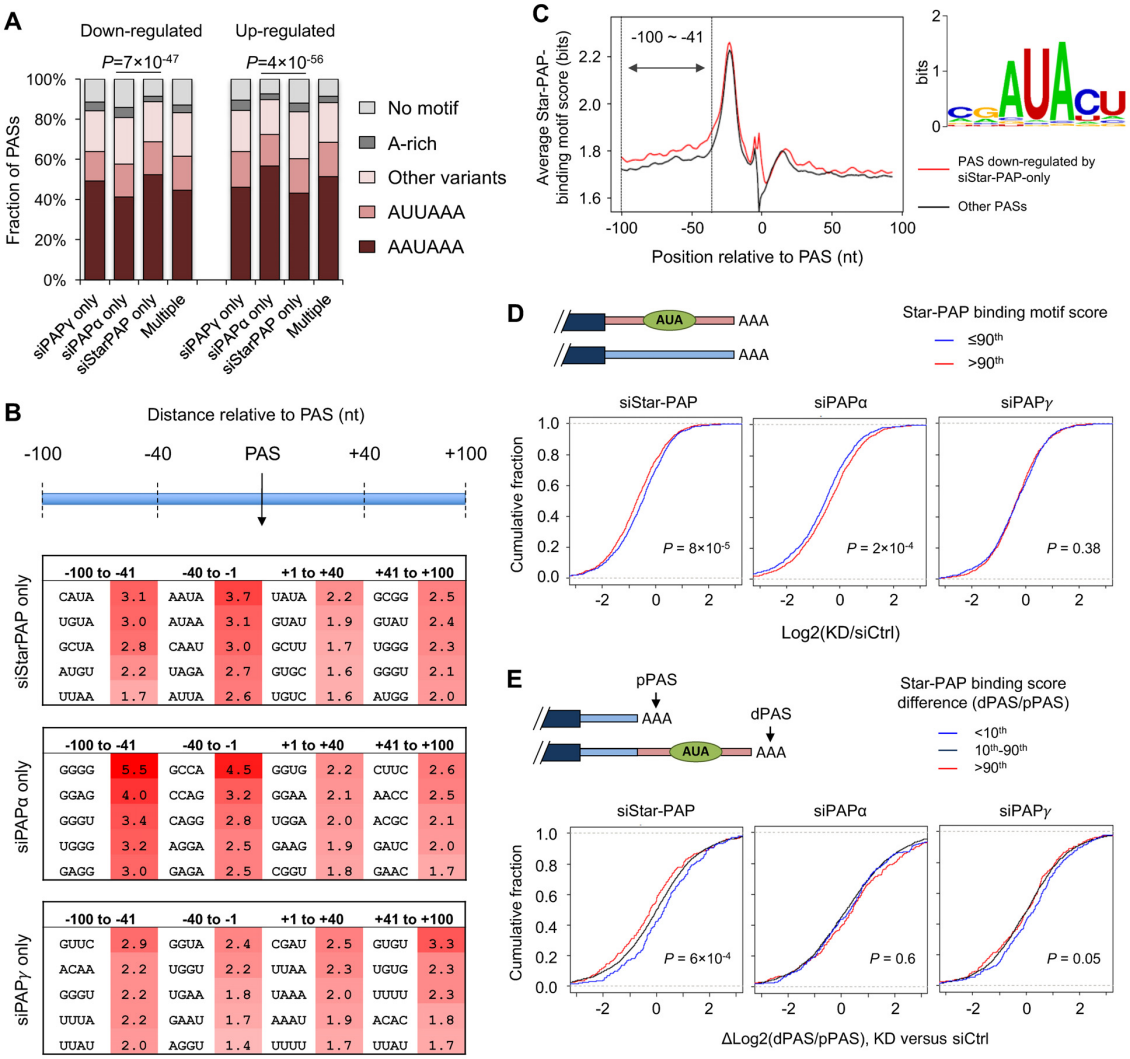
Sequence motifs associated with Star-PAP-dependent PAS usage. (**A**) Polyadenylation signal AAUAAA and its variants in the PASs that were commonly or uniquely regulated by the three PAPs. Other variants are 10 single nucleotide variants reported in (38) and A-rich motif was defined in (62). ‘Multiple’ stands for the PAS group commonly regulated by all three PAPs. Chi-square test *P*-values between siStarPAP-only and siPAPα-only groups were indicated. (**B**) Tetramers enriched for sequences around the PASs regulated by different PAPs. Numbers in the table were –log_10_ (*P*-value) (Fisher's Exact Test, see ‘Materials and Methods’ section for details). Four regions around the PAS were analyzed as indicated at the top. (**C**) Star-PAP binding motifs around the PAS. Left, Average Star-PAP binding motif scores around the PASs whose corresponding transcripts were downregulated by siStar-PAP only (red line) and other PASs (black line) that were not affect by KD of Star-PAP. Right, the Star-PAP binding motif sequence logo. (**D**) Cumulative distribution function (CDF) curve of log2Ratio of transcripts with predicted Star-PAP binding in three KD samples. Those with highly predicted Star-PAP binding were scored above the 90th percentile (red line) in the −100 to −41 nt region upstream of the PAS, while the target binding motif scores ≤90th (blue line) represent lower binding affinity. *P*-values (based on K-S test) comparing two PAS groups were indicated in the plots. (**E**) CDF curve of PAS relative expression difference (RED, see ‘Materials and Methods’ section for detail) between distal and proximal 3′UTR-PASs for three groups of genes, which were selected based on the difference in predicted Star-PAP binding between distal and proximal PAS isoforms. The Star-PAP binding prediction was based on the averaged motif score in the −100 to −41 nt region upstream of the PAS. *P*-values (K-S test) were indicated for the difference between the blue and red lines, corresponding to the <10th percentile and >90th percentile sets, respectively.

In order to further differentiate the PAPs on their selective usage of the PASs, we investigated whether there are sequence features around the PASs that could potentially contribute to their recognition by different PAPs. Interestingly, the PASs downregulated by Star-PAP KD tended to be associated with the canonical CPA signal AAUAAA more frequently than those downregulated by PAPα KD (Figure 3A and Supplementary Figure S4), suggesting that different sequence motifs around the PAS may be preferred by different PAPs. To address this more thoroughly, we analyzed all 4-mers enriched for regions surrounding regulated PASs. We found that the downregulated PASs by different PAP deficiencies displayed quite distinct 4-mer enrichments (Figure 3B). For example, the PASs downregulated only by siStar-PAP were enriched with motifs such as CAUA, UGUA, GCUA in the −100 to −41 nt region and AAUA, AUAA, CAAU in the −40 to −1 nt region (Figure 3B). This result is consistent with the observation that the PASs with the canonical AAUAAA motif tended to be downregulated by siStar-PAP (Figure 3A). By contrast, the PASs downregulated only by siPAPα showed enrichment of G-rich motifs (GGGG, GGAG, GGGU) and GCCA in the two upstream regions, respectively (Figure 3B). These results suggest that sequence motifs around a PAS play a role in determining PAS selection by the different PAPs.

We and others recently reported a characteristic AUA element within a GC-rich sequence background for Star-PAP binding to RNA (12,39). To score the AUA element within the transcripts of the PAP-target genes, we specifically examined transcripts containing this motif in the −100 to +100 nt region of PAS that were affected by the downregulation of the PAPs (Figure 3C). Interestingly, the transcripts downregulated by siStar-PAP only showed stronger signals of this motif in the −100 to −41 nt region upstream of their PASs compared to other transcripts (Figure 3C). We therefore specifically scored each transcript based on the average matching score of the motif in the −100 to −41 nt region, and compared transcript expression in different KD samples. We found that the transcripts with high scores (top 10%) tended to be significantly downregulated after Star-PAP KD (Figure 3D) as compared to other transcripts (*P* = 8 × 10^−5^, K-S test). In contrast, this trend was not observed in PAPα or PAPγ KDs (Figure 3D).

We further compared two 3′UTR-APA isoforms from each gene for their relative abundance changes after the PAP KDs. Based on the difference in the AUA element score, we divided genes into three groups (Figure 3E, upper panel). Significantly, the gene group whose distal PAS isoform had a higher score than the proximal PAS isoform by the greatest margin (top 10%) tended to show the most significant downregulation of the distal PAS isoform relative to the proximal PAS isoform (Figure 3E). This trend was significantly more conspicuous in the siStar-PAP samples, but not seen with siPAPα or siPAPγ samples. These data further support the notion that transcripts with the AUA motif upstream of the PAS are highly dependent on Star-PAP for expression, suggesting a direct, sequence-dependent role of Star-PAP in the control of PAS usage.

### Star-PAP governs the expression of several key mRNAs and lncRNAs

**Figure 4.**
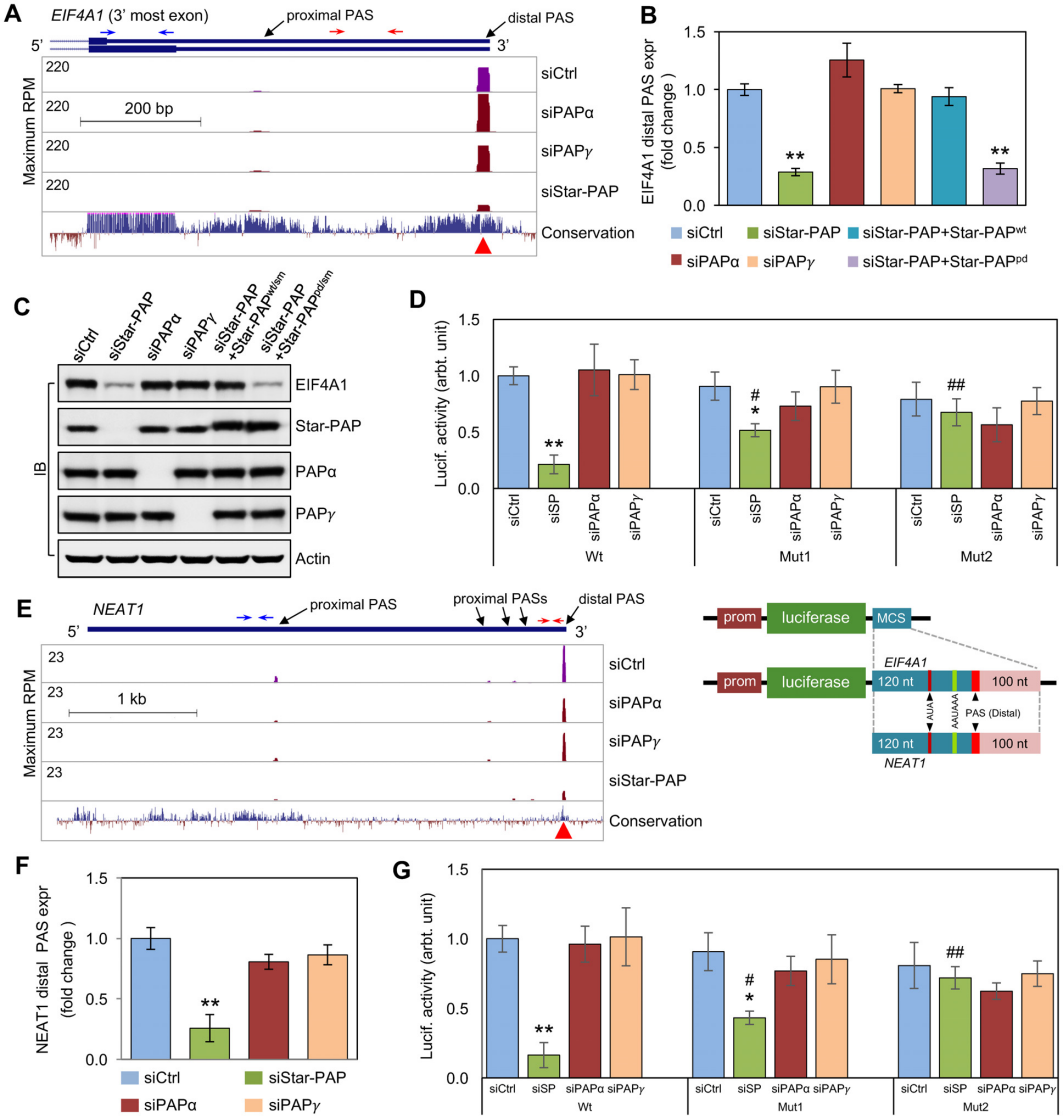
Star-PAP regulates APA of select protein-coding and lncRNA genes. (**A**) 3′READS detected marked changes in the *EIF4A1* mRNA isoform expression corresponding to the distal PAS after siRNA knockdown of Star-PAP, PAPα, or PAPγ. The PAP regulation of the expression of the *EIF4A1* mRNA isoform generated using the distal PAS (**B**) and the protein expression (**C**) was evaluated using the knockdown and rescue experiments in HEK293 cells. (**D**) Luciferase reporter assays with the *EIF4A1* distal PAS-flanking sequences that carry the wt, mutant AUA (Mut1) or mutant AUA and AAUAAA (Mut2). The bottom panel depicts the luciferase reporter constructs that were integrated into the pLightSwitch-3′UTR vector. (**E**) The effect of the PAP knockdowns on the usage of the *NEAT1* RNA PASs was detected by the 3′READS. (**F**) Star-PAP-specific regulation of the distal PAS usage was confirmed by qRT-PCR. (**G**) Luciferase reporter assays with the *NEAT1* distal PAS-flanking sequences that carry the wt, mutant AUA (Mut1) or mutant AUA and AAUAAA (Mut2). The luciferase reporter constructs were illustrated in the lower panel of (D). The primers for the relative expression of the mRNA isoform containing the distal PAS over the reference sequence of the *EIF4A1* and *NEAT1* RNAS were indicated by red and blue arrows, respectively, in (A) and (E). Error bars represent mean ± s.d. of three independent experiments with triplicates for each experimental condition. *P < 0.01 and **P < 0.001 represent the significance of the mean of three independent experiments relative to the control, and ^#^*P* < 0.01 and ^##^*P* < 0.001 were for the significance of the mean of three independent experiments relative to the siStar-PAP of the wt group based on one-way analysis of variance (One-way ANOVA) using StatPlus (AnalystSoft).

To validate our genomic findings, several genes that displayed APA changes were selected for additional analysis. EIF4A1 is a helicase that unwinds the 5′UTR of mRNA for the initiation of protein translation, and is involved in embryogenesis (40) and certain disease conditions, such as cancer (41). The regulation of *EIF4A1* expression is unknown. Our 3′READS data demonstrated that two PASs were detected within the 3′UTR of *EIF4A1* mRNA (Figure 4A and Supplementary Figure S5A). Downregulation of Star-PAP, but not PAPα or PAPγ, greatly reduced the usage of the *EIF4A1* distal PAS, which has an upstream AUA motif (Figure 4A and Supplementary Figure S5A). Consistent with the 3′READS data, quantitative real-time reverse transcription PCR (qRT-PCR, Figure 4B) and IB (Figure 4C) analyses showed that Star-PAP knockdown diminished the expression of the *EIF4A1* relative transcript corresponding to the distal 3′UTR sequence and the protein levels, which could be rescued by the wt Star-PAP but not the polymerase-dead (pd) mutant, both of which carried silent mutations (Star-PAP^wt/sm^ and Star-PAP^pd/sm^) that are insensitive to the siRNA KD of Star-PAP (Figure 4C and D).

To investigate whether the AUA motif mediates Star-PAP usage of the distal PAS, we carried out luciferase reporter assays in HEK293 cells transfected with the 3′UTR reporter vector containing the wt (intact AUA) or the mutated AUA (Mut1: AUA > AAA) in the presence or absence of simultaneous AAUAAA mutation (Mut2: AUA > AAA and AAUAAA > AUUAAA; Figure 4D, lower panel and Supplementary Figure S6). The results showed that siRNA knockdown of Star-PAP markedly decreased the expression of the wt RNA sequence around the *EIF4A1* distal PAS as reflected by the reduced luciferase activities within the cells (Figure 4D). Mutation of the AUA motif alone recovered the luciferase expression to a large extent, while additional mutation of the polyadenylation signal to a less-preferred sequence for Star-PAP further reduced the effect of Star-PAP knockdown on the 3′UTR expression. In contrast, downregulation of PAPα or PAPγ had no significantly effects on the luciferase activities within the cells transfected with the wt or the mutant sequence motifs within the *EIF4A1* 3′UTR, although a trend of slight decrease in the UTR expression was observed in the mutant samples upon PAPα or PAPγ knockdown (Figure 4D).

The nuclear paraspeckle assembly transcript 1 (*NEAT1*) RNA is a polyadenylated lncRNA transcribed by the RNA polymerase II. NEAT1 serves as a critical component and is essential for the formation of the paraspeckle structure in the nucleus (42). *NEAT1* expression is required for human mammary gland development and lactation (43), and is associated with cancer progression (44). Five PASs in the *NEAT1* gene were detected by our 3′READS data (Figure 4E). Interestingly, the distal PAS was downregulated by siStar-PAP (Figure 4E and Supplementary Figure S5B). The preferred usage of *NEAT1* distal PAS by Star-PAP was corroborated by qRT-PCR analysis using primers specific for the transcripts using the last PAS relative to the upstream first PAS expression (Figure 4F). Interestingly, we also found the AAUAAA polyA signal and a AUA sequence element upstream of the *NEAT1* distal PAS (Supplementary Figure S5). To test the function of these sequence elements, as described above for *EIF4A1*, Luciferase reporter assays using the cellular expression of the reporter vectors carrying the RNA sequence that contains the distal PAS of *NEAT1* with wt or mutant AUA or/and AAUAAA motifs were performed (Figure 4G and Supplementary Figure S6). Star-PAP-dependent regulation of the reporter gene expression, which associated with the existence of the wt or/and the mutant RNA motifs, was observed similar to that of the experiments for the *EIF4A1* 3′UTR mini gene expression (Figure 4G). The luciferase assay data indicate that both the AUA and the AAUAAA motifs are involved in Star-PAP-mediated gene expression, and Star-PAP selectively uses the distal PAS of *EIF4A1 and NEAT1* for the generation of their mRNAs. In the case of *EIF4A1*, its major mRNA isoform that was processed by Star-PAP using the distal PAS accounts for the cellular EIF4A1 protein levels.

Additionally, the Star-PAP-specific control of distal APA isoforms was corroborated also with several other genes, including chromatin assembly factor 1 subunit A (*CHAF1A*) and cytohesin 2 (*CYTH2*) (Supplementary Figure S7). For *EIF4A1* and *NEAT1*, the sequences around the distal PAS were found to be associated with Star-PAP but not PAPα and PAPγ as demonstrated by RNA immunoprecipitation (RIP, Supplementary Figure S8). In contrast, the 3′UTR of the Star-PAP non-target *GCLC* mRNA displayed association with PAPα, and to a much less but detectable extent, with PAPγ (Supplementary Figure S8). Taken together, these data collectively indicate that Star-PAP regulates the expression of both mRNAs and lncRNAs.

### Star-PAP regulates the expression of select *PTEN* APA isoforms that control its mRNA and protein levels

PTEN plays fundamental roles in many cellular functions and human health (45–48). Genetic loss or mutations of *PTEN* are key determinants in the incidence and penetrance of cancers (49,50). Subtle reduction in PTEN protein levels enhances tumor progression (51). Transcriptional regulation of *PTEN* expression by transcription factors has been extensively studied (48,52), and microRNA (miRNA)-mediated repression of its mRNA has also been implicated in its control (46,49,53).

**Figure 5.**
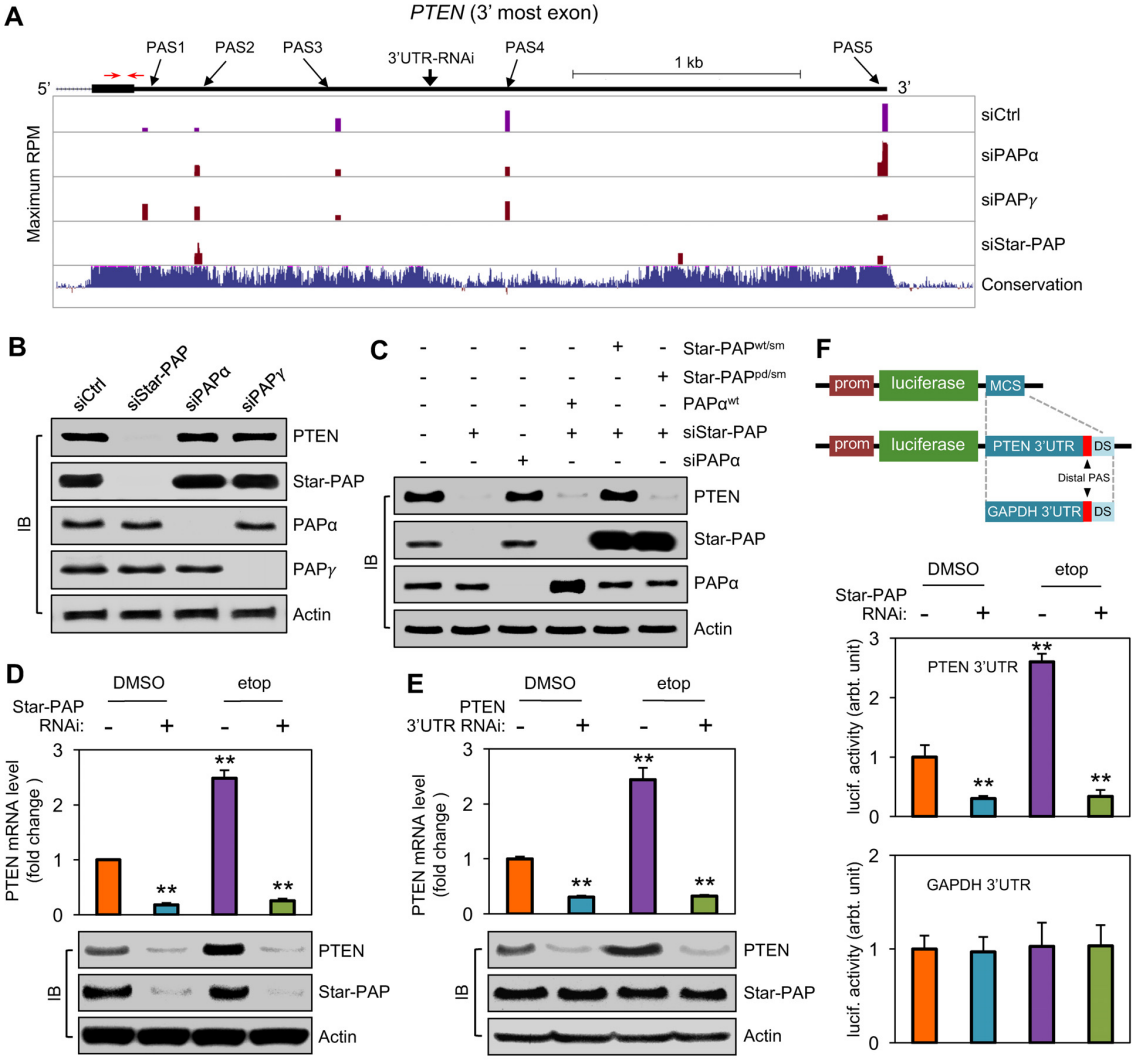
Selective usage of PAS within *PTEN* 3′UTR by Star-PAP controls cellular PTEN levels in response to DNA damage. (**A**) PAS distribution along the *PTEN* mRNA 3′UTR and the expression changes of the detected PASs after KD of the nuclear PAPs. PAS IDs and the location of the PTEN siRNA target site were indicated. (**B**) PTEN protein level changes after knocking down the individual PAPs in HEK293 cells. (**C**) Knockdown and rescue assay. No rescue of the PTEN protein expression was discerned with PAPα and Star-PAP^pd/sm^ ectopic expression after Star-PAP KD. In contrast, Star-PAP^wt/sm^ expression restored PTEN levels. (**D**) *PTEN* mRNA and protein levels in the cells with or without Star-PAP KD and in the presence or absence of DNA damage induction by etoposide treatment were examined by qRT-PCR (upper panel) and IB (lower panels). DMSO was used as vehicle control for the treatment. qRT-PCR primer pairs indicated with red arrows in (A) are located in the CDS region of the *PTEN* gene. (**E**) *PTEN* mRNA 3′UTR-specific KD reduced both basal and DNA damage-induced PTEN expression as measured by qRT-PCR for mRNA (upper panel) and IB for protein (lower panel) levels. The siRNA targeting site within the *PTEN* mRNA 3′UTR between PAS3 and PAS4 is shown in (A). (**F**) Analysis of *PTEN* 3′UTR expression by luciferase reporter assays. The 3′UTR of *PTEN* or *GAPDH* mRNA was subcloned into the pLightSwitch_3′UTR reporter vector for expression assessment in HEK293 cells using ELISA. The relative luciferase activities were normalized to the mock treatment control. Error bars represent standard error of the means of three independent experiments with triplicates for each experimental condition. Prom = promoter; MCS = multiple cloning site; DS = downstream sequence. The Error bars in (D), (E) and (F) represent mean ± s.d. of three independent experiments with triplicates for each experimental condition. **P < 0.001: significance relative to the control with one-way analysis of variance (one-way ANOVA).

Multiple *PTEN* mRNA isoforms had been reported in human cells (54). However, it is unclear how APA plays a role in generating these isoforms. Our 3′READS data showed that at least five PASs were detectible within the 3′UTR of the *PTEN* mRNA (Figure 5A). KD of the three nuclear PAPs caused usage changes of different PASs to variable degrees (Figure 5A and Supplementary Figure S9). Whereas PAPα KD only slightly reduced the proximal PAS usage and siPAPγ minimally affected the PAS usage profile, Star-PAP KD markedly decreased most of the distal, especially the third to fifth, PAS usages (Figure 5A). RIP analysis exhibited that Star-PAP, but not PAPα or PAPγ, associated with the *PTEN* 3′UTR around the distal PAS (Supplementary Figure S8). These data indicate that the PAPs potentially mediate APA of the *PTEN* mRNA isoforms. Importantly, while PAPα and PAPγ KDs had no detectable impacts on the PTEN protein levels, Star-PAP KD markedly diminished the amount of the cellular PTEN (Figure 5B).

The specificity of Star-PAP polyadenylation of *PTEN* mRNA and the corresponding protein expression was further examined using a KD/rescue approach, where the endogenous Star-PAP or canonical PAP was knocked down by siRNAs, and Star-PAP^wt/sm^, Star-PAP^pd/sm^ or PAPα^wt^/PAPγ^wt^ was ectopically expressed in the cells. While KD of Star-PAP but not PAPα or PAPγ downregulated PTEN protein levels, expression of Star-PAP^wt/sm^ restored PTEN levels (Figure 5C). Expression of Star-PAP^pd/sm^, PAPα^wt^ and PAPγ^wt^ (not shown) failed to rescue the reduction of PTEN proteins. These data further support the notion that Star-PAP, but not the canonical PAPs, controls PTEN expression through regulation of the distal PASs.

Since PTEN expression is induced by stress signals including DNA damage (46,48,55), and Star-PAP could mediate 3′ end processing of certain stress-response genes (9,11–13), we next examined whether Star-PAP regulates gene expression in cells experiencing DNA damage induced by etoposide. Using qRT-PCR and IB, we found that etoposide treatment increased PTEN protein levels in dose- and time-dependent manners (Supplementary Figure S10A and B), which also corresponded to enhanced *PTEN* mRNA expression (Figure 5D). siStar-PAP not only decreased the basal PTEN levels as shown in Figure 5B but also ablated the DNA damage-augmented *PTEN* mRNA and protein expression (Figure 5D). Surprisingly, when we downregulated the previously identified nuclear signaling cascades that mediate the DNA damage and oxidative stress responses by knocking down the key components, PKCα, PIPKIα and CKIα/ε, PTEN protein levels were not affected (Supplementary Figure S10C and D). These results demonstrate that Star-PAP regulates APA in a signaling- and target gene-specific manner.

The significance of the Star-PAP-dependent expression of distal PAS isoforms of *PTEN* was also assessed using siRNA targeting the 3′UTR sequence of *PTEN* between PAS3 and PAS4 (Figure 5A). The siRNA, which could only affect the expression of the transcript isoforms with sequences downstream of PAS3, and had no overlap with any known miRNA targeting sites, dramatically reduced the overall *PTEN* mRNA and protein expression (Figure 5E), the effect of which was similar to that of Star-PAP KD (Figure 5E versus B and D). These data indicated that the long *PTEN* mRNA isoforms generated via APA appear to be the major transcripts responsible for PTEN protein production.

To further examine whether Star-PAP-mediated regulation of *PTEN* isoforms is at the 3′ end, a luciferase reporter system was used, where the full-length *PTEN* 3′UTR with 132 nt of extended sequence downstream of PAS 5 was inserted into the luciferase reporter vector (Figure 5F, upper panel). The construct was then expressed in HEK293 cells followed by etoposide treatment in the presence or absence of siStar-PAP. The results showed that the luciferase activity was enhanced in response to etoposide stimulation. But both the basal and the stimulated luciferase activities were ablated upon Star-PAP KD (Figure 5F). This resembles the *PTEN* regulation by Star-PAP and further indicates that the 3′ end sequences of the transcripts are sufficient for the regulation. In contrast, the luciferase activities for the control construct containing the 3′UTR of *GAPDH*, which did not show regulation by Star-PAP, were not affected by either etoposide treatment or KD of Star-PAP. These data highlight the importance of Star-PAP-mediated APA in the regulation of PTEN expression, which has important roles in cellular stress response and many other biological functions.

## DISCUSSION

**Figure 6.**
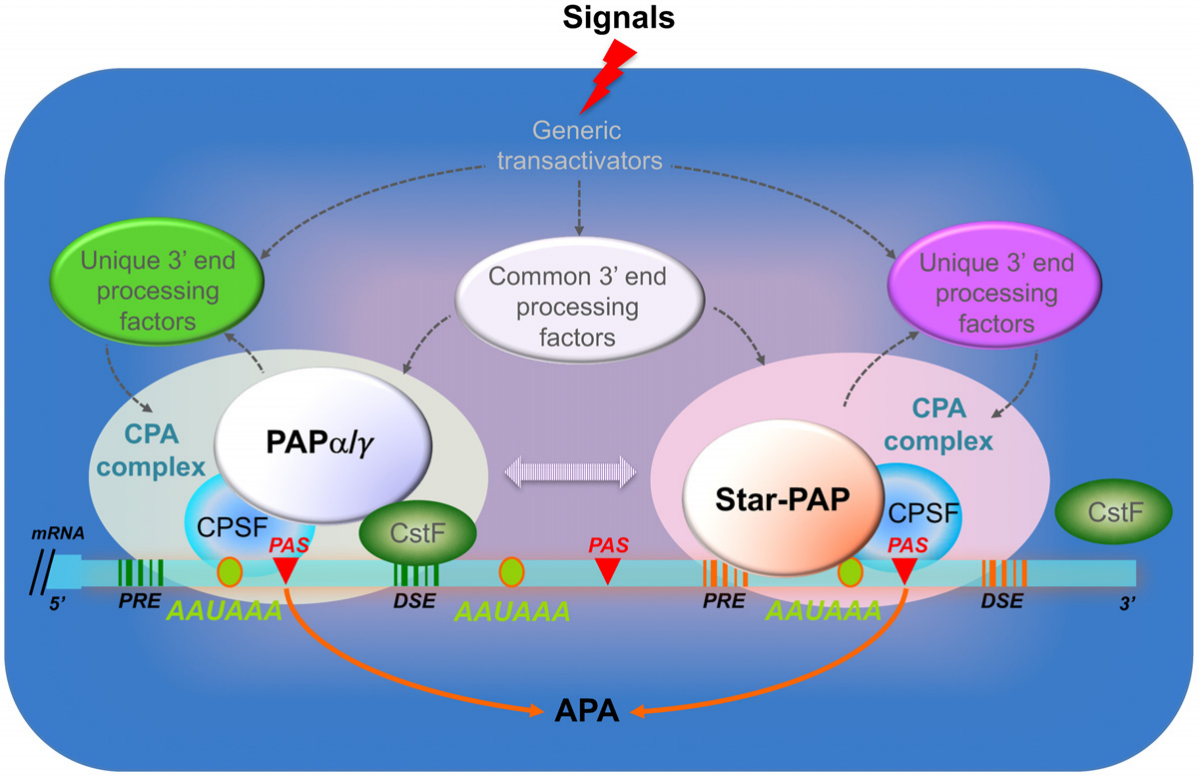
Model of APA regulation by the nuclear non-canonical and canonical PAPs. Cellular signals stimulate certain transactivators, which further activate specific 3′ end processing factors that selectively integrate into distinct mRNA 3′ end cleavage and polyadenylation (CPA) complexes for the activation of Star-PAP or the canonical PAPs. The PAP recognition element (PRE) upstream of PAS serves as a docking site for specific RNA-binding proteins including Star-PAP. In contrast, recruitment of PAPα or PAPγ to the CPA complex requires CstF64 binding to the downstream sequence element (DSE) and its interaction with the CPSF processing factors. Substitution of a specific PAP within an mRNA 3′ end processing complex by another PAP could potentially be permissible for the processing of certain but not all target genes. Solid arrows indicate direct regulation; dotted arrows represent observations from previous studies or suggested regulatory mechanisms.

Our work has delineated a PAS-centric gene expression paradigm controlled by the nuclear PAPs (Figure 6). In this model, PAS regulation by PAPs involves both common and unique 3′ end processing factors that are modulated by upstream generic transactivators in response to cellular signals, resulting in APA or/and changes in the expression levels of gene transcripts. The specificity of each individual PAP toward a target RNA sequence is associated with the distinct features of a PAS, such as the PAS location within the sequence, and its surrounding sequence motifs.

In addition to the RNA sequence determinants, our data also indicate that the PAPs play key roles in PAS selection, impacting APA genome wide. While the three PAPs appear to influence comparably large sets of overlapping PASs, highlighting the importance of cellular PAP activities for gene expression, each individual PAP modulates the usage of a specific group of PASs. Interestingly, Star-PAP-dependent PASs displayed more biased features than those for the other two PAPs analyzed. Deficient Star-PAP expression caused significant downregulation of a number of distal PASs with a concomitant upregulation of intronic PASs. The PASs regulated by PAPα, however, showed an opposite trend. This result implies that the PAS position in a gene is an important parameter governing the type of PAP used for the RNA 3′ end processing. Additionally, the presence of AUA-containing sequence motifs in the upstream region of Star-PAP-regulated PASs further implicates direct interactions between Star-PAP and RNA substrates. This result is in agreement with our previous studies indicating that Star-PAP-mediated 3′ end processing minimally requires only the CPSF subcomplex, and PAPα- and PAPγ-mediated processing may additionally require the CstF subcomplex (12). It has not been clear, however, whether the PAS location bias is coupled with or independent of the sequence motif for Star-PAP regulation. Using the luciferase mini gene constructs containing the wt and mutant AUA or/and AAUAAA motifs upstream of the distal PAS of the *EIF4A1* or *NEAT1* RNA, we have revealed that the optimal Star-PAP binding to and processing of its target RNAs minimally require the existence of both the intact AUA and AAUAAA motifs. The large but not full restoration of the expression of the *EIF4A1* or *NEAT1* reporter genes that had mutations in both of the motifs after Star-PAP knockdown suggests that either the AUUAAA polyadenylation signal still supports Star-PAP interaction with the RNAs and processing of the transcripts to some extent or additional sequence elements around the PAS coordinates the processing in a minor way (Figure 4B and F). These additional elements could be the GC-rich sequences up- and down-stream of the PAS as we previously reported (12).

Notably, we found that the PASs with high Star-PAP binding potentials, judging from the motif score, tend to be more conserved in surrounding sequences than those with low binding potentials (Supplementary Figure S11), highlighting the functional importance of the AUA motif and the Star-PAP usage of critical PASs. Of the two model genes by which we examined the relationship of the presence of a functional AUA element with Star-PAP-mediated PAS usage, *EIF4A1* has an AUA element conserved across multiple species (Supplementary Figure S5A, bottom panel) while the AUA sequence upstream of the *NEAT1* distal PAS is only present in human (Supplementary Figure S5B, bottom panel). On this note, it is worth mentioning that, of the three PAPs analyzed in this study, Star-PAP appears to be the most recently evolved PAP as its domain structure is only conserved in vertebrates whereas PAPα and PAPγ are widely conserved in eukaryotes. In terms of how the emergence of Star-PAP in evolution plays a role in 3′UTR evolution and gene regulation, and whether Star-PAP similarly mediates distal PAS usage through selective usage of the AUA or/and AAUAAA motifs in other species, need to be examined in the future.

The impact of deficient PAP functions on gene expression suggests that the polyadenylation activity might be a rate-limiting step for RNA expression of many genes. We did not find a correlation between gene expression change and APA regulation (Supplementary Figure S12), suggesting that gene expression regulation in general is not due to APA isoforms having different mRNA stabilities. Thus, how exactly gene expression is affected by the PAPs is still an open question that requires further investigation. One possibility is that the poly(A) tail length, which is relevant to mRNA stability, might be regulated by the PAPs. Using the poly(A) tail length data of HEK293T cells that were previously generated by the Bartel *et al.* (56), we specifically compared the poly(A) tail length for PASs affected by different PAPs (Supplementary Figure S13). We found that PASs that are specifically regulated by Star-PAP appear to have slightly shorter poly(A) tails than those regulated by other PAPs. This result suggests that poly(A) tail length control may potentially be a mechanism by which PAPs regulate gene expression. Future studies, however, are needed to unravel the details of the tripartite interactions between PAS, poly(A) tail and PAP.

An additional level of the target gene-specific PAP regulation may be the integration of the generic transactivators, such as PIPKIα, CKIα/ε and PKCδ (9,11,12), and unique mRNA 3′ end processing factors (Figure 6). This is supported by a recent study on the APA regulation by cytoplasmic polyadenylation element binding protein 1, which shuttled into the nucleus and recruited CPSF to an alternative PAS, where the complex could interfere with splicing machinery and affect the levels of splicing factors (57). Consistently, studies in yeast showed that kinases and RNA processing factors localized dynamically to mRNA–protein complexes during stress response (58).

An important case in point in this study is APA regulation of the *PTEN* gene expression. It appears that Star-PAP, PAPα and PAPγ are all involved in mediating the expression of different *PTEN* mRNA isoforms (Figure 5A). However, only Star-PAP appears to be essential for the production of the long *PTEN* mRNA isoforms by using the distal PASs. Since PAPα and PAPγ were not able to rescue the reduction of PTEN expression induced by Star-PAP KD, our result supports non-redundant affinities of PAPs toward different *PTEN* APA sites. Moreover, the usage of the distal *PTEN* PASs is stimulated by DNA damage signals, which also depends on Star-AP. Interestingly, counter to previous reports indicating that mRNA isoforms generated using proximal PAS produce more proteins (23,24,59), we found that *PTEN* mRNA isoforms derived from the use of distal PASs are responsible for production of the majority of the protein (Figure 5). This appears to be also true for the translation elongation factor EIF4A1 (Figure 4A–C). Future studies are needed to elucidate how 3′UTR length and/or specific 3′UTR elements contribute to the regulation of gene expression. Nevertheless, shortening of the *PTEN* mRNA 3′ UTR is consistent with the general shortening of 3′UTRs in proliferating or cancer cells (23,24). In addition, our data showing that the long APA isoforms of *PTEN* rather than the short ones are chiefly responsible for PTEN protein expression highlight the complexity of the impact of APA on gene expression. Presumably, mRNA stability and its translation into protein are selectively dependent on the specific properties of the 3′UTR sequence, instead of the UTR length *per se* (60,61).

In summary, this study depicts a PAP-medicated intricate network, which impacts APA and RNA expression genome-wide and indicates that the RNA 3′ end-mediated control of gene expression is as important and complex as that at the 5′ end.

## ACCESSION NUMBER

The 3′READS data reported in this paper have been deposited in the NCBI database GEO (Accession Number: GSE84461).

## Supplementary Material

Supplementary Data

## References

[1] Proudfoot N.J. Transcriptional termination in mammals: stopping the RNA polymerase II juggernaut. Science. 2016; 352:aad9926. 27284201 10.1126/science.aad9926 PMC5144996

[2] Colgan D.F., Manley J.L. Mechanism and regulation of mRNA polyadenylation. Genes Dev. 1997; 11:2755–2766. 9353246 10.1101/gad.11.21.2755

[3] Zheng D., Tian B. Sizing up the poly(A) tail: insights from deep sequencing. Trends Biochem. Sci. 2014; 39:255–257. 24751511 10.1016/j.tibs.2014.04.002 PMC4369379

[4] Sachs A., Wahle E. Poly(A) tail metabolism and function in eucaryotes. J. Biol. Chem. 1993; 268:22955–22958. 8226806

[5] Shi Y., Manley J.L. The end of the message: multiple protein-RNA interactions define the mRNA polyadenylation site. Genes Dev. 2015; 29:889–897. 25934501 10.1101/gad.261974.115 PMC4421977

[6] Topalian S.L., Kaneko S., Gonzales M.I., Bond G.L., Ward Y., Manley J.L. Identification and functional characterization of neo-poly(A) polymerase, an RNA processing enzyme overexpressed in human tumors. Mol. Cell. Biol. 2001; 21:5614–5623. 11463842 10.1128/MCB.21.16.5614-5623.2001 PMC87282

[7] Yang Q., Nausch L., Martin G., Keller W., Doublie S. Crystal structure of human Poly(A) polymerase gamma reveals a conserved catalytic core for canonical Poly(A) polymerases. J. Mol. Biol. 2013; 426:43–50. 24076191 10.1016/j.jmb.2013.09.025 PMC3878066

[8] Lee Y.J., Lee Y., Chung J.H. An intronless gene encoding a poly(A) polymerase is specifically expressed in testis. FEBS Lett. 2000; 487:287–292. 11150526 10.1016/s0014-5793(00)02367-x

[9] Gonzales M.L., Mellman D.L., Anderson R.A. CKIalpha is associated with and phosphorylates star-PAP and is also required for expression of select star-PAP target messenger RNAs. J. Biol. Chem. 2008; 283:12665–12673. 18305108 10.1074/jbc.M800656200 PMC2431003

[10] Laishram R.S., Anderson R.A. The poly A polymerase Star-PAP controls 3′-end cleavage by promoting CPSF interaction and specificity toward the pre-mRNA. EMBO J. 2010; 29:4132–4145. 21102410 10.1038/emboj.2010.287 PMC3018792

[11] Laishram R.S., Barlow C.A., Anderson R.A. CKI isoforms alpha and epsilon regulate Star-PAP target messages by controlling Star-PAP poly(A) polymerase activity and phosphoinositide stimulation. Nucleic Acids Res. 2011; 39:7961–7973. 21729869 10.1093/nar/gkr549 PMC3185439

[12] Li W., Laishram R.S., Ji Z., Barlow C.A., Tian B., Anderson R.A. Star-PAP control of BIK expression and apoptosis is regulated by nuclear PIPKIalpha and PKCdelta signaling. Mol. Cell. 2012; 45:25–37. 22244330 10.1016/j.molcel.2011.11.017 PMC3268557

[13] Mellman D.L., Gonzales M.L., Song C., Barlow C.A., Wang P., Kendziorski C., Anderson R.A. A PtdIns4,5P2-regulated nuclear poly(A) polymerase controls expression of select mRNAs. Nature. 2008; 451:1013–1017. 18288197 10.1038/nature06666

[14] Wahle E. Purification and characterization of a mammalian polyadenylate polymerase involved in the 3′ end processing of messenger RNA precursors. J. Biol. Chem. 1991; 266:3131–3139. 1993684

[15] Murthy K.G., Manley J.L. The 160-kD subunit of human cleavage-polyadenylation specificity factor coordinates pre-mRNA 3′-end formation. Genes Dev. 1995; 9:2672–2683. 7590244 10.1101/gad.9.21.2672

[16] Elkon R., Ugalde A.P., Agami R. Alternative cleavage and polyadenylation: extent, regulation and function. Nat. Rev. Genet. 2013; 14:496–506. 23774734 10.1038/nrg3482

[17] Lutz C.S., Moreira A. Alternative mRNA polyadenylation in eukaryotes: an effective regulator of gene expression. WIREs RNA. 2010; 2:23–31. 10.1002/wrna.47 PMC3029013 21278855

[18] Shi Y. Alternative polyadenylation: new insights from global analyses. RNA. 2012; 18:2105–2117. 23097429 10.1261/rna.035899.112 PMC3504663

[19] Di Giammartino D.C., Nishida K., Manley J.L. Mechanisms and consequences of alternative polyadenylation. Mol. Cell. 2011; 43:853–866. 21925375 10.1016/j.molcel.2011.08.017 PMC3194005

[20] Tian B., Manley J.L. Alternative polyadenylation of mRNA precursors. Nat. Rev. Mol. Cell Biol. 2017; 18:18–30. 27677860 10.1038/nrm.2016.116 PMC5483950

[21] Zhang H., Lee J.Y., Tian B. Biased alternative polyadenylation in human tissues. Genome biology. 2005; 6:R100. 16356263 10.1186/gb-2005-6-12-r100 PMC1414089

[22] Smibert P., Miura P., Westholm J.O., Shenker S., May G., Duff M.O., Zhang D., Eads B.D., Carlson J., Brown J.B. Global patterns of tissue-specific alternative polyadenylation in Drosophila. Cell Rep. 2012; 1:277–289. 22685694 10.1016/j.celrep.2012.01.001 PMC3368434

[23] Sandberg R., Neilson J.R., Sarma A., Sharp P.A., Burge C.B. Proliferating cells express mRNAs with shortened 3′ untranslated regions and fewer microRNA target sites. Science. 2008; 320:1643–1647. 18566288 10.1126/science.1155390 PMC2587246

[24] Mayr C., Bartel D.P. Widespread shortening of 3′UTRs by alternative cleavage and polyadenylation activates oncogenes in cancer cells. Cell. 2009; 138:673–684. 19703394 10.1016/j.cell.2009.06.016 PMC2819821

[25] Ji Z., Lee J.Y., Pan Z., Jiang B., Tian B. Progressive lengthening of 3′ untranslated regions of mRNAs by alternative polyadenylation during mouse embryonic development. Proc. Natl. Acad. Sci. U.S.A. 2009; 106:7028–7033. 19372383 10.1073/pnas.0900028106 PMC2669788

[26] Shepard P.J., Choi E.A., Lu J., Flanagan L.A., Hertel K.J., Shi Y. Complex and dynamic landscape of RNA polyadenylation revealed by PAS-Seq. RNA. 2011; 17:761–772. 21343387 10.1261/rna.2581711 PMC3062186

[27] Tian B., Manley J.L. Alternative cleavage and polyadenylation: the long and short of it. Trends Biochem. Sci. 2013; 38:312–320. 23632313 10.1016/j.tibs.2013.03.005 PMC3800139

[28] Li W., You B., Hoque M., Zheng D., Luo W., Ji Z., Park J.Y., Gunderson S.I., Kalsotra A., Manley J.L. Systematic profiling of poly(A)+ transcripts modulated by core 3′ end processing and splicing factors reveals regulatory rules of alternative cleavage and polyadenylation. PLoS Genet. 2015; 11:e1005166. 25906188 10.1371/journal.pgen.1005166 PMC4407891

[29] Yao C., Choi E.A., Weng L., Xie X., Wan J., Xing Y., Moresco J.J., Tu P.G., Yates J.R. 3rd, Shi Y. Overlapping and distinct functions of CstF64 and CstF64tau in mammalian mRNA 3′ processing. RNA. 2013; 19:1781–1790. 24149845 10.1261/rna.042317.113 PMC3884657

[30] de Klerk E., Venema A., Anvar S.Y., Goeman J.J., Hu O., Trollet C., Dickson G., den Dunnen J.T., van der Maarel S.M., Raz V. Poly(A) binding protein nuclear 1 levels affect alternative polyadenylation. Nucleic Acids Res. 2012; 40:9089–9101. 22772983 10.1093/nar/gks655 PMC3467053

[31] Jenal M., Elkon R., Loayza-Puch F., van Haaften G., Kuhn U., Menzies F.M., Oude Vrielink J.A., Bos A.J., Drost J., Rooijers K. The poly(A)-binding protein nuclear 1 suppresses alternative cleavage and polyadenylation sites. Cell. 2012; 149:538–553. 22502866 10.1016/j.cell.2012.03.022

[32] Gunderson S.I., Polycarpou-Schwarz M., Mattaj I.W. U1 snRNP inhibits pre-mRNA polyadenylation through a direct interaction between U1 70K and poly(A) polymerase. Mol. Cell. 1998; 1:255–264. 9659922 10.1016/s1097-2765(00)80026-x

[33] Kaida D., Berg M.G., Younis I., Kasim M., Singh L.N., Wan L., Dreyfuss G. U1 snRNP protects pre-mRNAs from premature cleavage and polyadenylation. Nature. 2010; 468:664–668. 20881964 10.1038/nature09479 PMC2996489

[34] Flavell S.W., Kim T.K., Gray J.M., Harmin D.A., Hemberg M., Hong E.J., Markenscoff-Papadimitriou E., Bear D.M., Greenberg M.E. Genome-wide analysis of MEF2 transcriptional program reveals synaptic target genes and neuronal activity-dependent polyadenylation site selection. Neuron. 2008; 60:1022–1038. 19109909 10.1016/j.neuron.2008.11.029 PMC2630178

[35] Berg M.G., Singh L.N., Younis I., Liu Q., Pinto A.M., Kaida D., Zhang Z., Cho S., Sherrill-Mix S., Wan L. U1 snRNP determines mRNA length and regulates isoform expression. Cell. 2012; 150:53–64. 22770214 10.1016/j.cell.2012.05.029 PMC3412174

[36] Devany E., Park J.Y., Murphy M.R., Zakusilo G., Baquero J., Zhang X., Hoque M., Tian B., Kleiman F.E. Intronic cleavage and polyadenylation regulates gene expression during DNA damage response through U1 snRNA. Cell Discov. 2016; 2:16013. 27462460 10.1038/celldisc.2016.13 PMC4906801

[37] Hoque M., Ji Z., Zheng D., Luo W., Li W., You B., Park J.Y., Yehia G., Tian B. Analysis of alternative cleavage and polyadenylation by 3′ region extraction and deep sequencing. Nat. Methods. 2013; 10:133–139. 23241633 10.1038/nmeth.2288 PMC3560312

[38] Tian B., Hu J., Zhang H., Lutz C.S. A large-scale analysis of mRNA polyadenylation of human and mouse genes. Nucleic Acids Res. 2005; 33:201–212. 15647503 10.1093/nar/gki158 PMC546146

[39] Ray D., Kazan H., Cook K.B., Weirauch M.T., Najafabadi H.S., Li X., Gueroussov S., Albu M., Zheng H., Yang A. A compendium of RNA-binding motifs for decoding gene regulation. Nature. 2013; 499:172–177. 23846655 10.1038/nature12311 PMC3929597

[40] Pause A., Sonenberg N. Mutational analysis of a DEAD box RNA helicase: the mammalian translation initiation factor eIF-4A. EMBO J. 1992; 11:2643–2654. 1378397 10.1002/j.1460-2075.1992.tb05330.x PMC556740

[41] Wolfe A.L., Singh K., Zhong Y., Drewe P., Rajasekhar V.K., Sanghvi V.R., Mavrakis K.J., Jiang M., Roderick J.E., Van der Meulen J. RNA G-quadruplexes cause eIF4A-dependent oncogene translation in cancer. Nature. 2014; 513:65–70. 25079319 10.1038/nature13485 PMC4492470

[42] Clemson C.M., Hutchinson J.N., Sara S.A., Ensminger A.W., Fox A.H., Chess A., Lawrence J.B. An architectural role for a nuclear noncoding RNA: NEAT1 RNA is essential for the structure of paraspeckles. Mol. Cell. 2009; 33:717–726. 19217333 10.1016/j.molcel.2009.01.026 PMC2696186

[43] Standaert L., Adriaens C., Radaelli E., Van Keymeulen A., Blanpain C., Hirose T., Nakagawa S., Marine J.C. The long noncoding RNA Neat1 is required for mammary gland development and lactation. RNA. 2014; 20:1844–1849. 25316907 10.1261/rna.047332.114 PMC4238351

[44] Chakravarty D., Sboner A., Nair S.S., Giannopoulou E., Li R., Hennig S., Mosquera J.M., Pauwels J., Park K., Kossai M. The oestrogen receptor alpha-regulated lncRNA NEAT1 is a critical modulator of prostate cancer. Nat. Commun. 2014; 5:5383. 25415230 10.1038/ncomms6383 PMC4241506

[45] Steck P.A., Pershouse M.A., Jasser S.A., Yung W.K., Lin H., Ligon A.H., Langford L.A., Baumgard M.L., Hattier T., Davis T. Identification of a candidate tumour suppressor gene, MMAC1, at chromosome 10q23.3 that is mutated in multiple advanced cancers. Nat. Genet. 1997; 15:356–362. 9090379 10.1038/ng0497-356

[46] Hollander M.C., Blumenthal G.M., Dennis P.A. PTEN loss in the continuum of common cancers, rare syndromes and mouse models. Nat. Rev. Cancer. 2011; 11:289–301. 21430697 10.1038/nrc3037 PMC6946181

[47] Oudit G.Y., Sun H., Kerfant B.G., Crackower M.A., Penninger J.M., Backx P.H. The role of phosphoinositide-3 kinase and PTEN in cardiovascular physiology and disease. J. Mol. Cell Cardiol. 2004; 37:449–471. 15276015 10.1016/j.yjmcc.2004.05.015

[48] Song M.S., Salmena L., Pandolfi P.P. The functions and regulation of the PTEN tumour suppressor. Nat. Rev. Mol. Cell Biol. 2012; 13:283–296. 22473468 10.1038/nrm3330

[49] Carracedo A., Alimonti A., Pandolfi P.P. PTEN level in tumor suppression: how much is too little. Cancer Res. 2011; 71:629–633. 21266353 10.1158/0008-5472.CAN-10-2488 PMC3249925

[50] Whang Y.E., Wu X., Suzuki H., Reiter R.E., Tran C., Vessella R.L., Said J.W., Isaacs W.B., Sawyers C.L. Inactivation of the tumor suppressor PTEN/MMAC1 in advanced human prostate cancer through loss of expression. Proc. Natl. Acad. Sci. U.S.A. 1998; 95:5246–5250. 9560261 10.1073/pnas.95.9.5246 PMC20246

[51] Alimonti A., Carracedo A., Clohessy J.G., Trotman L.C., Nardella C., Egia A., Salmena L., Sampieri K., Haveman W.J., Brogi E. Subtle variations in Pten dose determine cancer susceptibility. Nat. Genet. 2011; 42:454–458. 10.1038/ng.556 PMC3118559 20400965

[52] Tamguney T., Stokoe D. New insights into PTEN. J. Cell Sci. 2007; 120:4071–4079. 18032782 10.1242/jcs.015230

[53] Salmena L., Carracedo A., Pandolfi P.P. Tenets of PTEN tumor suppression. Cell. 2008; 133:403–414. 18455982 10.1016/j.cell.2008.04.013

[54] Hamilton J.A., Stewart L.M., Ajayi L., Gray I.C., Gray N.E., Roberts K.G., Watson G.J., Kaisary A.V., Snary D. The expression profile for the tumour suppressor gene PTEN and associated polymorphic markers. Br. J. Cancer. 2000; 82:1671–1676. 10817502 10.1054/bjoc.2000.1211 PMC2374512

[55] Ming M., He Y.Y. PTEN in DNA damage repair. Cancer Lett. 2012; 319:125–129. 22266095 10.1016/j.canlet.2012.01.003 PMC3326178

[56] Subtelny A.O., Eichhorn S.W., Chen G.R., Sive H., Bartel D.P. Poly(A)-tail profiling reveals an embryonic switch in translational control. Nature. 2014; 508:66–71. 24476825 10.1038/nature13007 PMC4086860

[57] Bava F.A., Eliscovich C., Ferreira P.G., Minana B., Ben-Dov C., Guigo R., Valcarcel J., Mendez R. CPEB1 coordinates alternative 3′-UTR formation with translational regulation. Nature. 2013; 495:121–125. 23434754 10.1038/nature11901

[58] Mitchell S.F., Jain S., She M., Parker R. Global analysis of yeast mRNPs. Nat. Struct. Mol. Biol. 2013; 20:127–133. 23222640 10.1038/nsmb.2468 PMC3537908

[59] Elkon R., Drost J., van Haaften G., Jenal M., Schrier M., Vrielink J.A., Agami R. E2F mediates enhanced alternative polyadenylation in proliferation. Genome Biol. 2012; 13:R59. 22747694 10.1186/gb-2012-13-7-r59 PMC3491381

[60] Atwater J.A., Wisdom R., Verma I.M. Regulated mRNA stability. Annu. Rev. Genet. 1990; 24:519–541. 2088178 10.1146/annurev.ge.24.120190.002511

[61] Casey J.L., Hentze M.W., Koeller D.M., Caughman S.W., Rouault T.A., Klausner R.D., Harford J.B. Iron-responsive elements: regulatory RNA sequences that control mRNA levels and translation. Science. 1988; 240:924–928. 2452485 10.1126/science.2452485

[62] Nunes N.M., Li W., Tian B., Furger A. A functional human Poly(A) site requires only a potent DSE and an A-rich upstream sequence. EMBO J. 2010; 29:1523–1536. 20339349 10.1038/emboj.2010.42 PMC2876958

