# Supplementary material for: Distinct regulation of alternative polyadenylation and gene expression by nuclear poly(A) polymerases
Source: Nucleic Acids Res. 2017 Jun 27;45(15):8930–42. doi: 10.1093/nar/gkx560 (PMC5587728; doi:10.1093/nar/gkx560)
Supplement: Supplementary Data [file gkx560_Supp.pdf]

Supplementary Material

Table of Contents

1. Supplementary Figures

Figure S1: Analysis of the efficiencies of the siRNA knockdowns

Figure S2: The expression profiles of mRNAs and lncRNAs in replicate samples

Figure S3: Global regulation of APA in different PAP KDs

Figure S4: Polyadenylation signal AAUAAA and its variants associated with the PASs that were commonly or uniquely regulated by the three PAPs

Figure S5: Star-PAP regulates *EIF4A* and *NEAT1* distal PAS expression

Figure S6: The wt and mutant RNA sequences of the model genes that were inserted into the pLightSwitch-3'UTR reporter vectors for luciferase assays

Figure S7: Star-PAP regulates *CHAF1A* and *CYTH2* distal PAS expression

Figure S8: RIP analysis of PAP association with the 3'UTR of the target mRNAs

Figure S9: The PASs and AUA motifs within the *PTEN* mRNA 3'UTR

Figure S10: PIPKIα, PKCδ and CKIα/ε do not regulate PTEN cellular levels under the experimental conditions

Figure S11: Conservation of PASs with different Star-PAP binding motif scores

Figure S12: Relationship between 3'UTR APA regulation and gene expression change

Figure S13: Poly(A) tail length for transcripts in different regulation groups after three PAP knockdowns

2. Supplementary Tables

Table S1: Mapping statistics of the RNA deep sequencing using 3'READS

Table S2: Gene Ontology groups associated with down-regulated genes after KD

Table S3: Gene Ontology terms enriched for genes with APA changes

3. Antibodies used in the study

4. Primers used for RT-PCR

5. siRNAs used in the experiments

6. References

1. Supplementary Figures

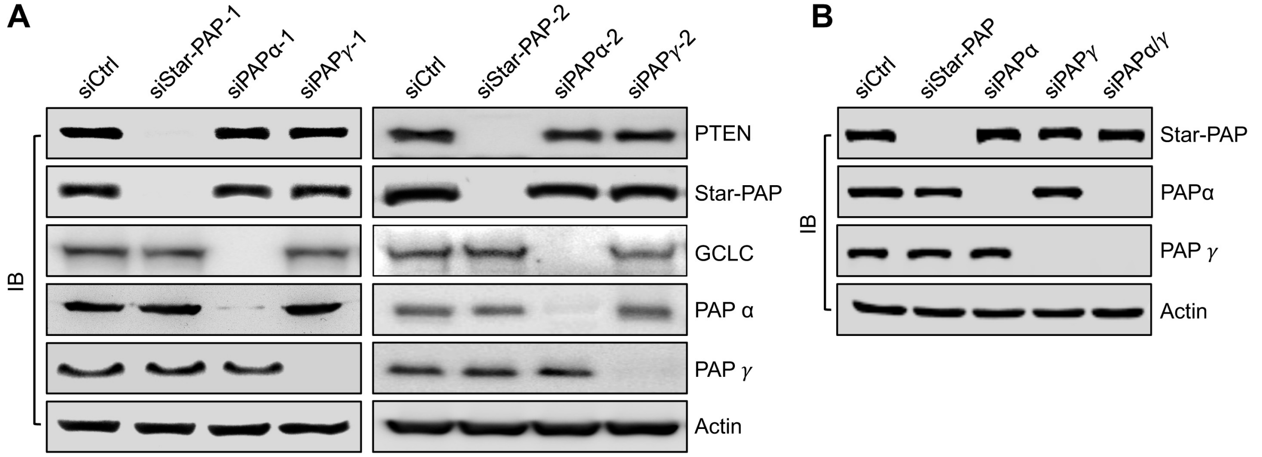

Figure S1. Analysis of the efficiencies of the siRNA knockdowns. (A) Analysis of potential off-target effects of the siRNAs using IB. **(B)** The effective knockdown of the PAPs by the siRNAs was evaluated by IB. Replicate cell samples were used for total RNA extraction followed by cDNA library preparation and 3'READS.

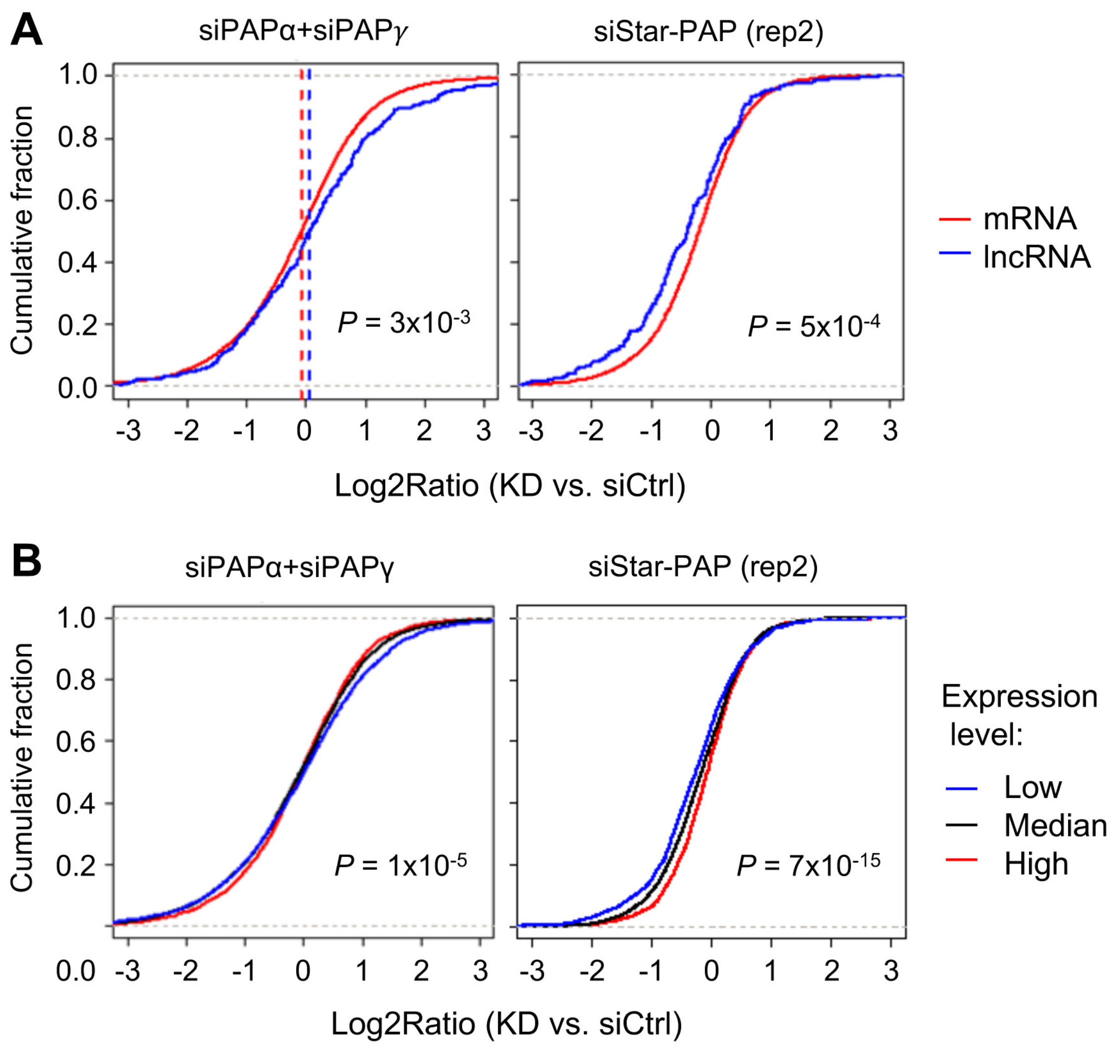

Figure S2. (A) The expression profiles of mRNAs and lncRNAs in replicate samples. (B) The expression levels of the different genes impacted by PAP knockdown. Low, median and high expression genes are those <25th, 25th-75th, >75th percentiles, respectively. *P*-value is based on K-S test comparing <25th with >75th groups.

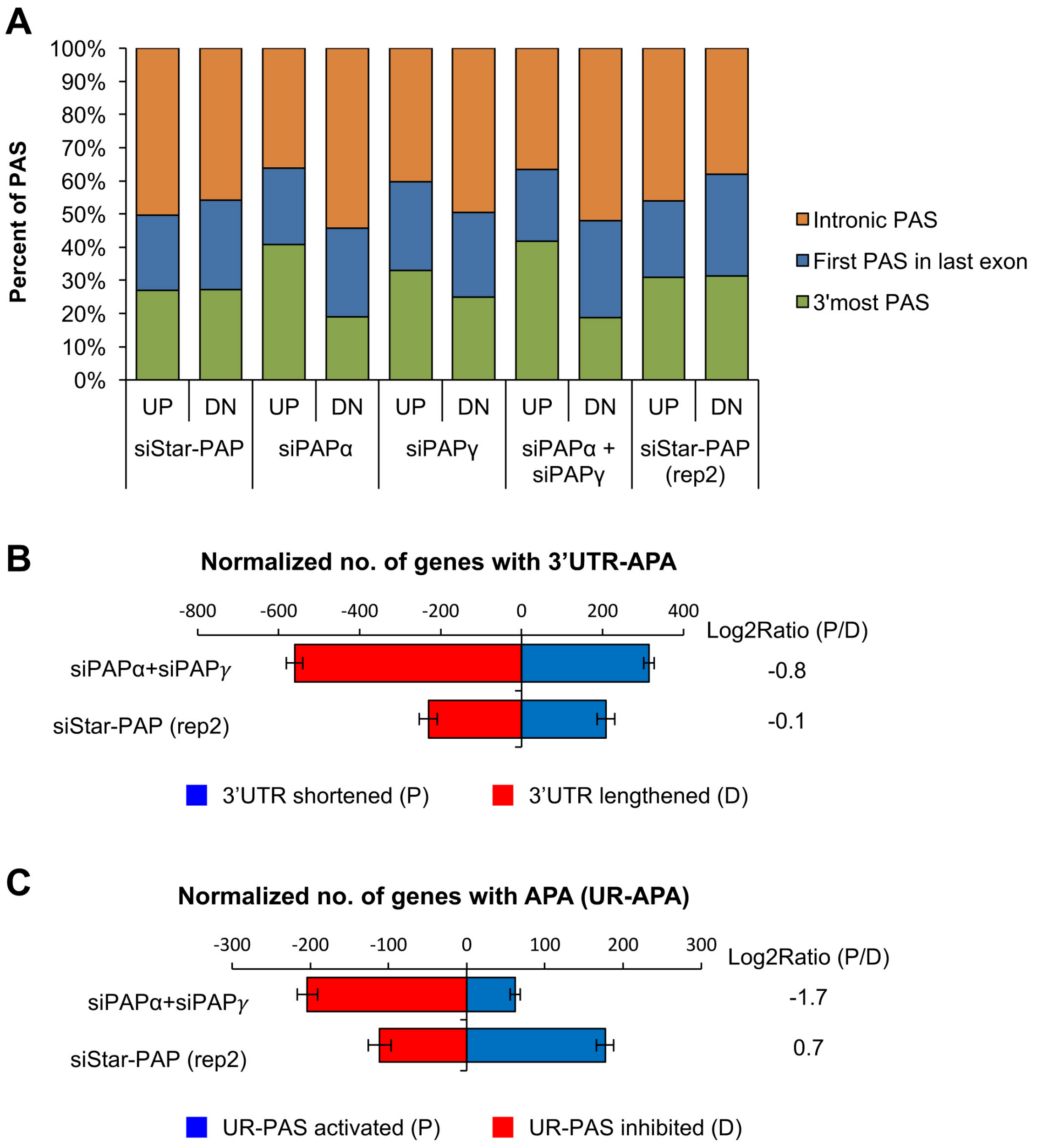

Figure S3. Global regulation of APA in different PAP KDs. (A) Percent of PAS in different locations of a gene upregulated (UP) or downregulated (DN) by different PAP knockdowns (FDR < 0.05, Significance Analysis of Alternative Polyadenylation, SAAP). **(B)** Regulation of 3’UTR-APA for PAPα+PAPγ double knockdown and siStar-PAP replicate 2 as analyzed by Global Analysis of Alternative Polyadenylation (GAAP). The log2Ratio of the number of genes with 3’UTR shortened (P for proximal PAS upregulated) to the number of genes with 3’UTR lengthened (D for distal PAS upregulated) is also shown. **(C)** Regulation of UR-APA for PAPα+PAPγ double knockdown and siStar-PAP replicate 2 as analyzed by GAAP. The log2Ratio of the number of genes with UR-PAS usage activated (P) to the number of genes with UR-PAS usage inhibited (D) is also shown.

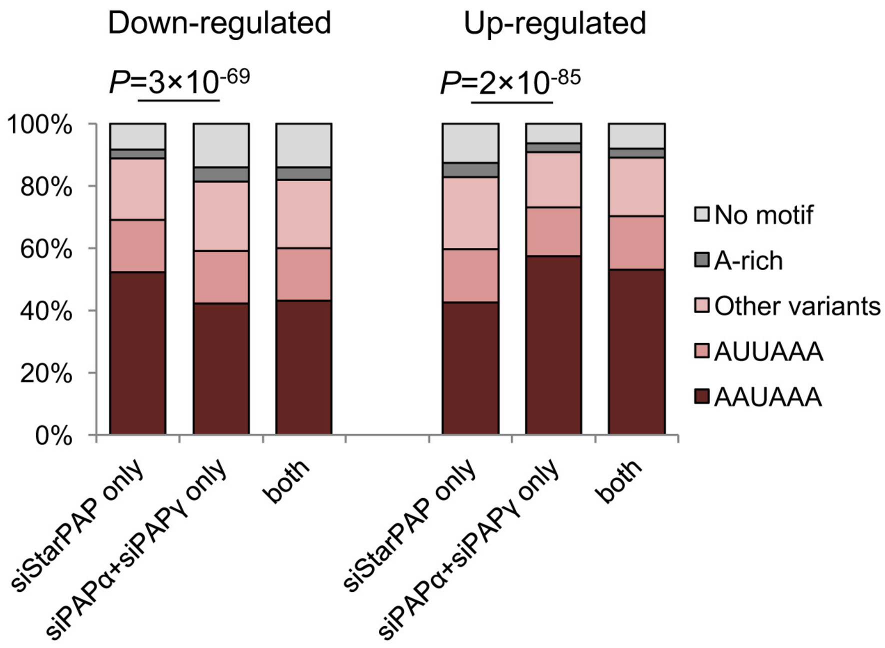

Figure S4. Polyadenylation signal AAUAAA and its variants associated with the PASs that were commonly or uniquely regulated by the three PAPs. As Figure 3A except that the PAPα and PAPγ double knockdown was compared with Star-PAP knockdown.

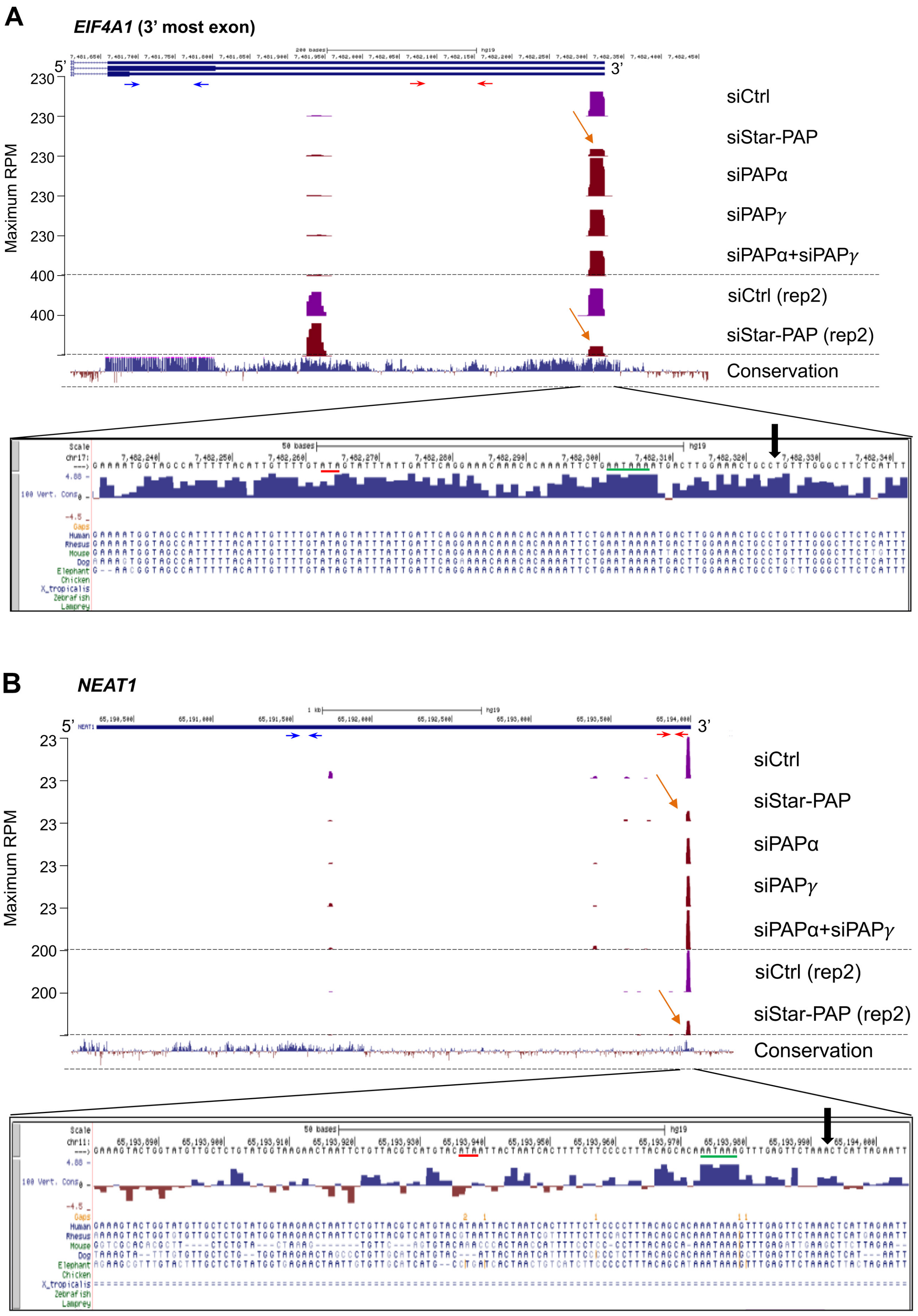
A
EIF4A1 (3' most exon)
5'
3'
Maximum RPM
230
230
230
230
230
400
400
siCtrl
siStar-PAP
siPAPα
siPAPγ
siPAPα+siPAPγ
siCtrl (rep2)
siStar-PAP (rep2)
Conservation
B
NEAT1
5'
3'
Maximum RPM
23
23
23
23
23
200
200
siCtrl
siStar-PAP
siPAPα
siPAPγ
siPAPα+siPAPγ
siCtrl (rep2)
siStar-PAP (rep2)
Conservation

Figure S5. Star-PAP regulates *EIF4A* and *NEAT1* distal PAS expression. (A) PAP regulation of the distal PAS usage within the 3'UTR *of EIF4A1* mRNA: Upper panel: 3'READS profile of the 3'-most exon of *EIF4A1*. The PAS downregulated after Star-PAP knockdown was indicated by orange arrow. The primer pairs used for the qRT-PCR analysis of the expression of the 3'-end sequences containing the distal PAS and a reference region were indicated by red and blue arrows, respectively. Lower panel: sequence conservation and multi-species sequence alignment of the PAS, which was downregulated after Star-PAP knockdown. The PAS was marked by an arrow. The polyadenylation signal and the potential Star-PAP binding motif AUA were underlined in green and red, respectively. **(B)** PAP regulation of the distal PAS usage within the *NEAT1* mRNA: Upper panel: 3'READS profile of the changes of the *NEAT1* PASs after KD of the PAPs. The PAS downregulated after Star-PAP knockdown was indicated by orange arrow. The primer pairs used for the qRT-PCR analysis of the expression of the 3'-end sequences containing the distal PAS and a reference region were indicated by red and blue arrows, respectively. Lower panel: sequence conservation and multi-species sequence alignment of the PAS, which was downregulated after Star-PAP knockdown. The PAS was marked by an arrow. The polyadenylation signal and the potential Star-PAP binding motif AUA were underlined in green and red, respectively.

EIF4A1 3’UTR sequence containing the distal PAS that was inserted into the pLightSwitch vector

GUCCCCAGGUGGGGGGAAGCAGGGGAGAGAAAAUGGUAGCCAUUUUUACAUUGUUUUGUAUAGUAUUUAUUGAUUC
AGGAAACAAACACAAAAUUCUGAAUAAAAUGACUUGGAAACUGC**C**UGUUUGGGCUUCUCAUUUCUUACCUCCCCUU
CCCUCUCCCACCUGCUACUGGGUGCAUCUCUGCUCCCCCCUUCCCCAGCAGAUGGUUACCUUUGGGCUG

NEAT1 RNA sequence containing the distal PAS that was inserted into the pLightSwitch vector

AAGUACUGGUAUGUUGCUCUGUAUGGUAAGAACUAAUUCUGUUACGUCAUGUACAUAAUUACUAAUCACUUUUCUU
CCCCUUUACAGCACAAAUAAAGUUUGAGUUCUAAACUCAUUAGA**A**UUGUUGUAUUGCUAUGUUACAUUUCUCGACC
CCUAUCACAUUGCCUUCAUAACGACUUUGGAUGUAUCUUCAUAUUGUAGAUUUAGGUCUAGAUUUGCUA

* The AUA motif, poly(A) signal, and polyadenylation site are highlighted in red.

Mutations for the luciferase assays shown in Figure 4B and 4F:

Wt: no change on sequence

Mut1: AUA > AAA

MUT2: AUA > AAA + AAUAAA > AUUAAA

Figure S6. The wt and mutant RNA sequences of the model genes that were inserted into the pLightSwitch-3’UTR reporter vectors for luciferase assays. Related to Fig. 4D and 4G.

A

***CHAF1A*: chromatin assembly factor 1, subunit A (p150)**

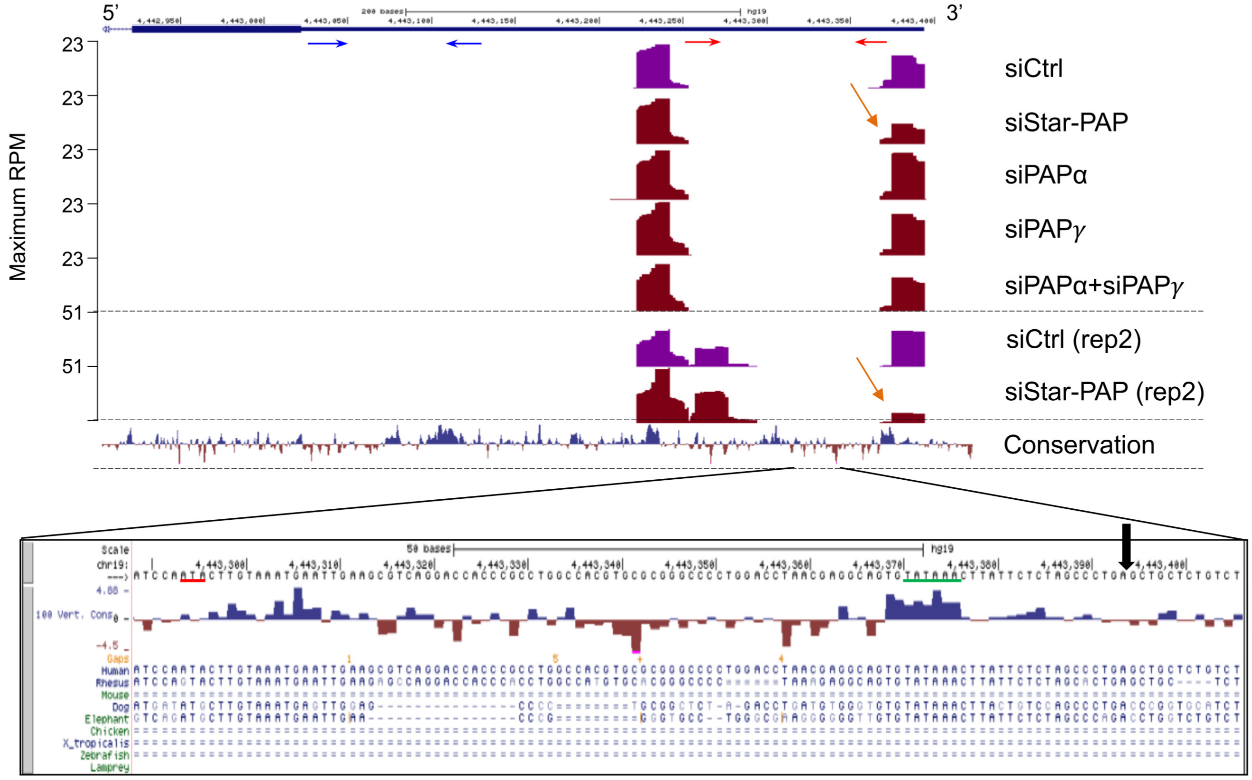

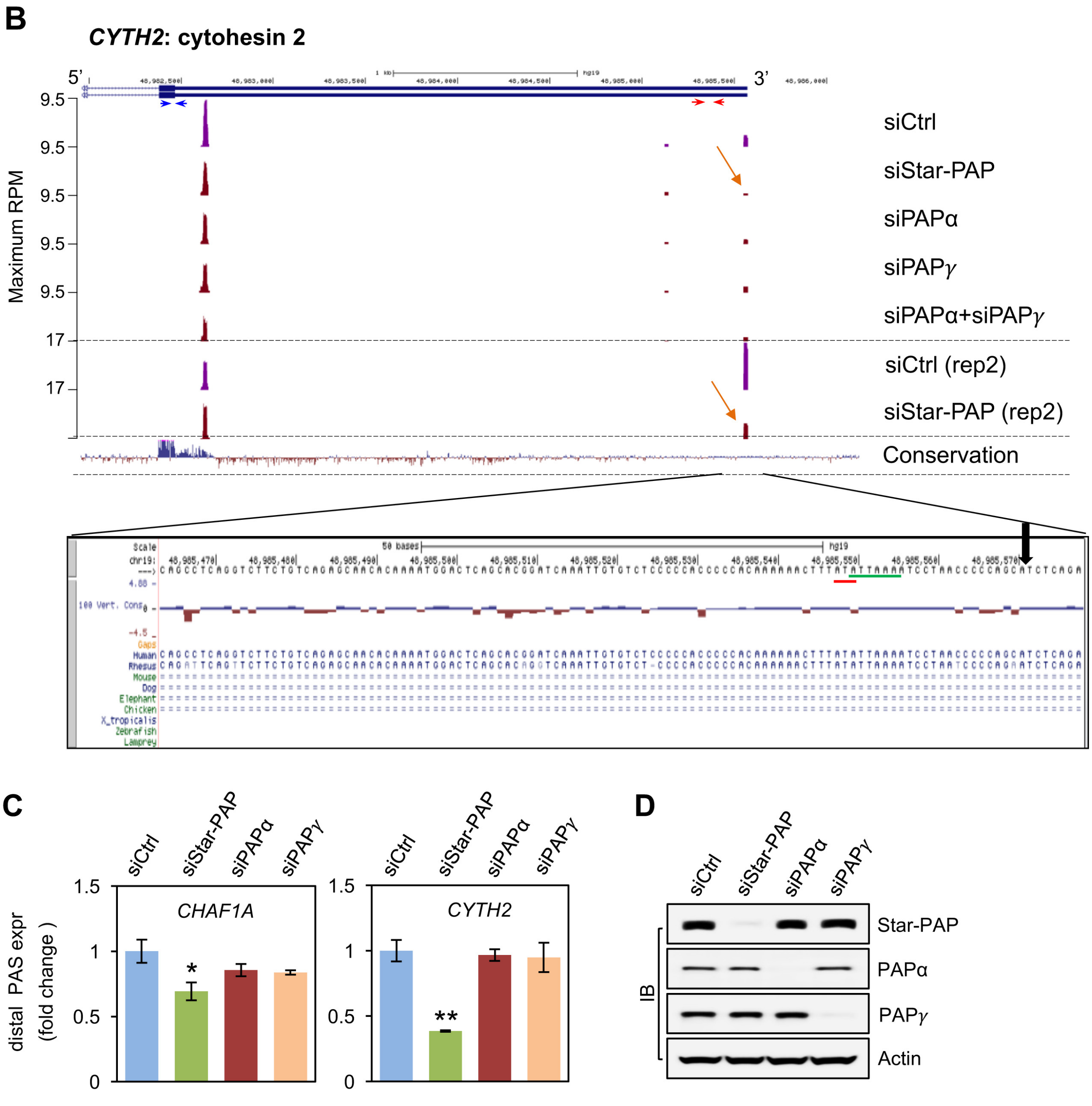
B
CYTH2: cytohesin 2
5'
3'
Maximum RPM
9.5
17
siCtrl
siStar-PAP
siPAPα
siPAPγ
siPAPα+siPAPγ
siCtrl (rep2)
siStar-PAP (rep2)
Conservation
C
siCtrl
siStar-PAP
siPAPα
siPAPγ
distal PAS expr
(fold change)
CHAF1A
CYTH2
1.5
1
0.5
0
*
**
D
IB
Star-PAP
PAPα
PAPγ
Actin

Figure S7. Star-PAP regulates *CHAF1A* and *CYTH2* distal PAS expression. (A) PAP regulation of the distal PAS usage within the 3'UTR *of CHAF1A* mRNA: Upper panel: 3'READS profile of the 3'-most exon of *CHAF1A*. The PAS downregulated after Star-PAP knockdown was indicated by orange arrow. The primer pairs used for the qRT-PCR analysis of the expression of the 3'-end sequences containing the distal PAS and a reference region were indicated by red and blue arrows, respectively. Lower panel: sequence conservation and multi-species sequence alignment of the PAS, which was downregulated after Star-PAP knockdown. The PAS was marked by an arrow. The polyadenylation signal and the potential Star-PAP binding motif AUA were underlined in green and red, respectively. **(B)** PAP regulation of the distal PAS usage within the 3'UTR of *CYTH2* mRNA: (A) Upper panel: 3'READS profile of the 3'-most exon of *CYTH2*. The PAS downregulated after Star-PAP knockdown was indicated by orange arrow. Lower panel: sequence conservation and multi-species sequence alignment of the PAS, which was downregulated after Star-PAP knockdown. The PAS was marked by an arrow. The polyadenylation signal and the potential Star-PAP binding motif AUA were underlined in green and red, respectively. **(C)** The relative expression of the mRNA isoform corresponding to the use of distal PAS over a reference region of *CHAF1A* and *CYTH2* by the individual PAPs, as examined via the siRNA knockdown approach, was analyzed using qRT-PCR. The primer pairs used for the qRT-PCR analysis of the expression of the 3'-end sequences containing the PAS and the reference region were indicated by red and blue arrows, respectively, in (A) and (B). Error bars represent mean ± s.d. of 3 independent experiments with triplicates for each experimental condition. *P<0.05 and **P<0.01 represent the significance of the mean of 3 independent experiments relative to the control. **(D)** The knockdown efficiencies of the PAPs by the siRNAs were evaluated by IB.

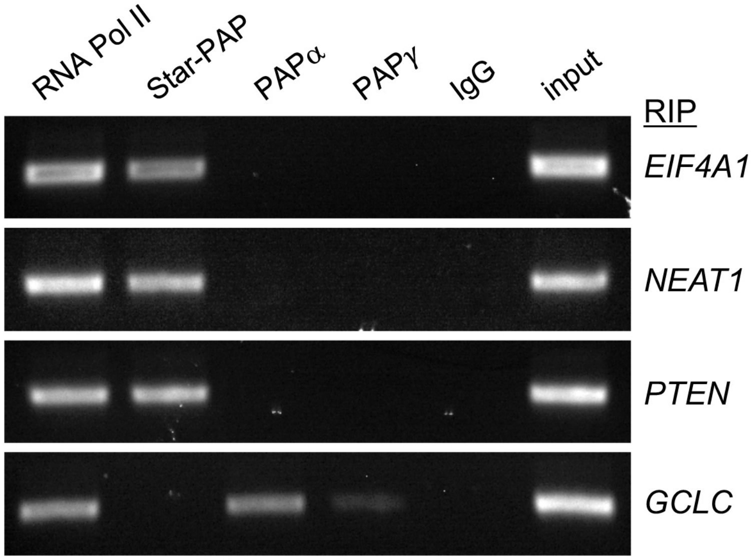

Figure S8. RIP analysis of PAP association with the 3'UTR of the target mRNAs. RNA Pol II and rabbit IgG were used as positive and negative IP controls, respectively. The sequences around the distal PAS were PCR amplified. The primers used for *EIF4A1* and *NEAT1* were the same as for the real-time qRT-PCR analysis of the relative PAS expression shown in Fig. 4, and those for *PTEN* were indicated in the Supplementary Data.

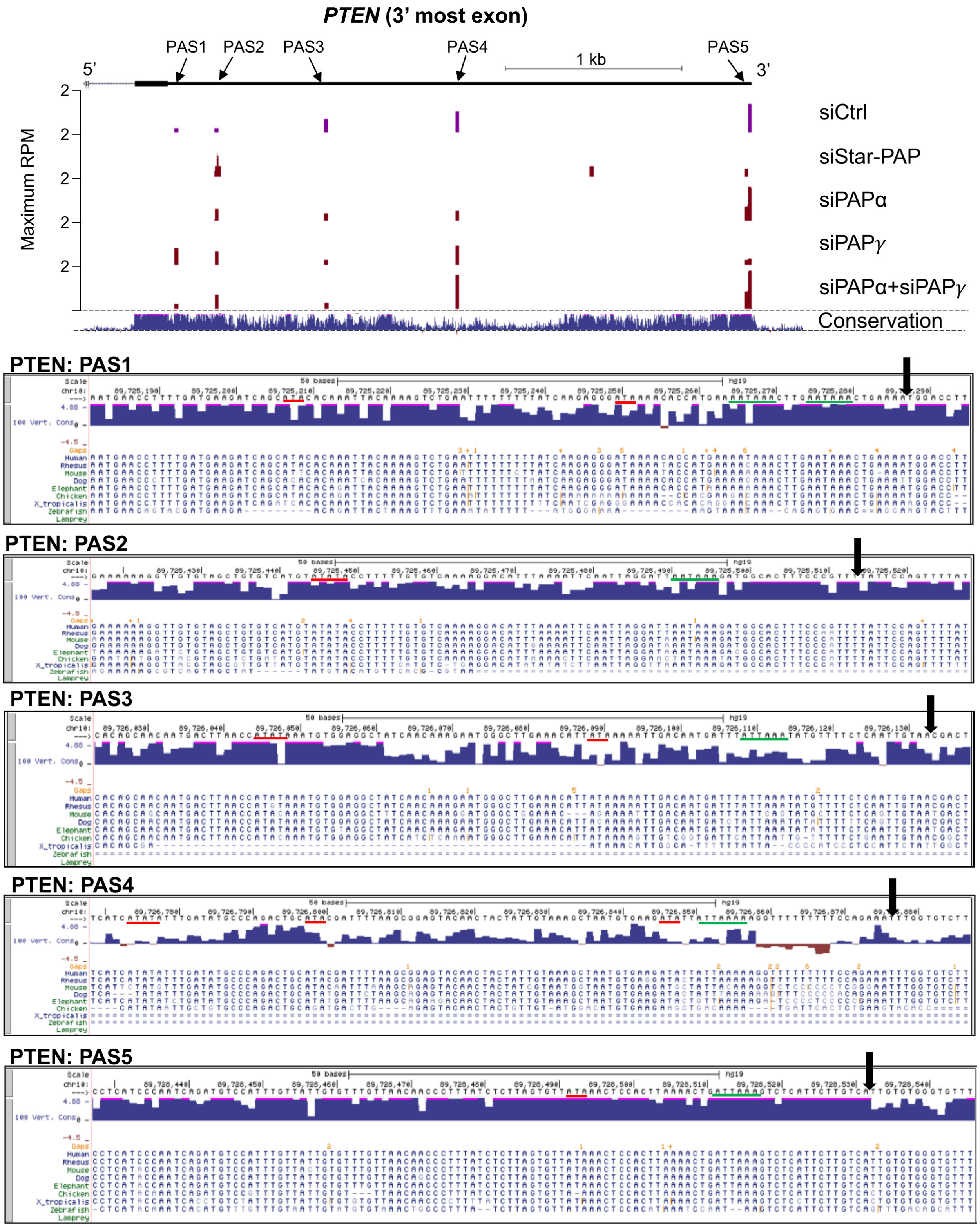

Figure S9. The PASs and AUA motifs within the *PTEN* mRNA 3'UTR. Upper panel: 3'READS profile of the 3'-most exon of *PTEN*. Lower panel: sequence conservation and multi-species sequence alignment of the PASs, which were marked by arrows. The poly(A) signals and the potential Star-PAP binding motifs AUA were underlined in green and red, respectively.

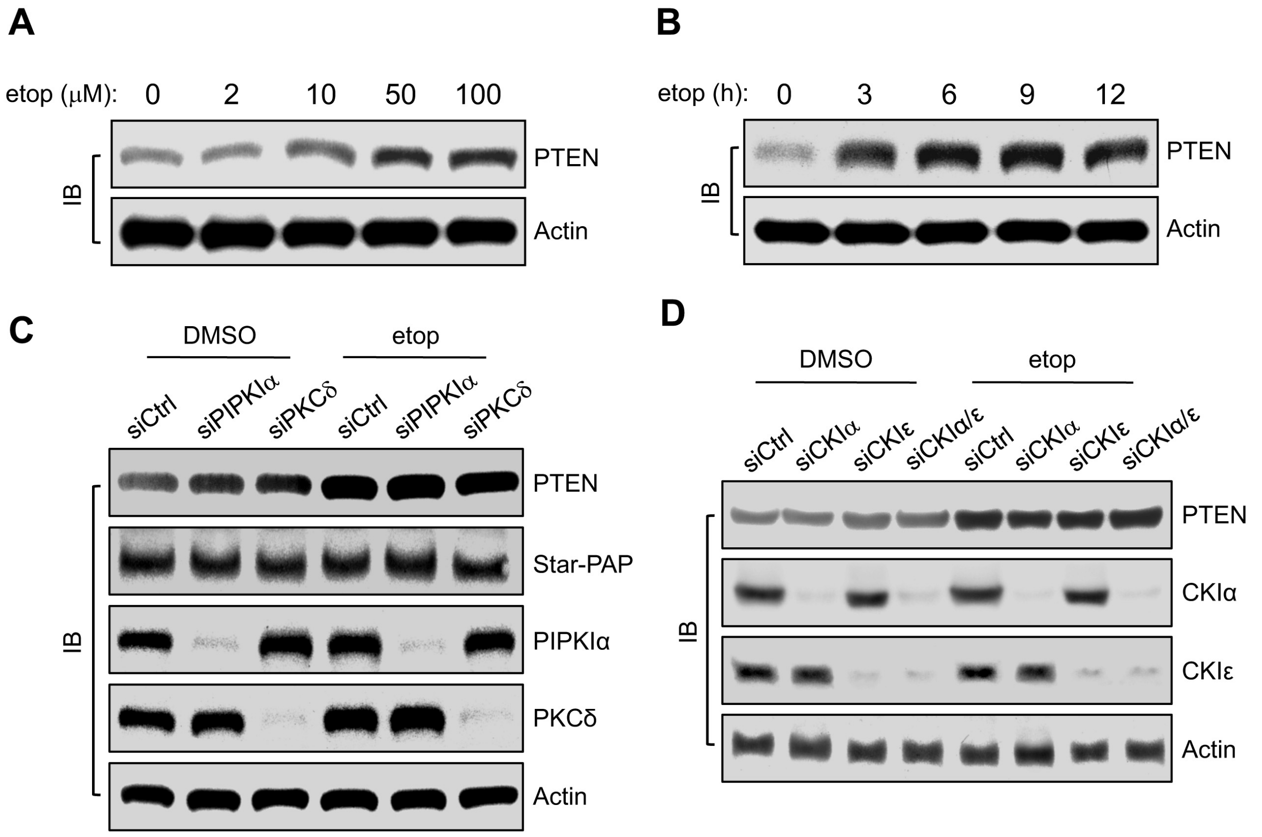

Figure S10: PIPKIα, PKCδ and CKIα/ε do not regulate PTEN cellular levels under the experimental conditions. IB results showed that the DNA damaging chemotherapeutic drug etoposide (A) dose- and (B) time-dependently induced PTEN protein expression. RNAi knockdown of PIPKIα or PKCδ (C) and CKIα or/and CKIε (D) in the presence or absence of etoposide had no effect on PTEN protein levels as detected by IB.

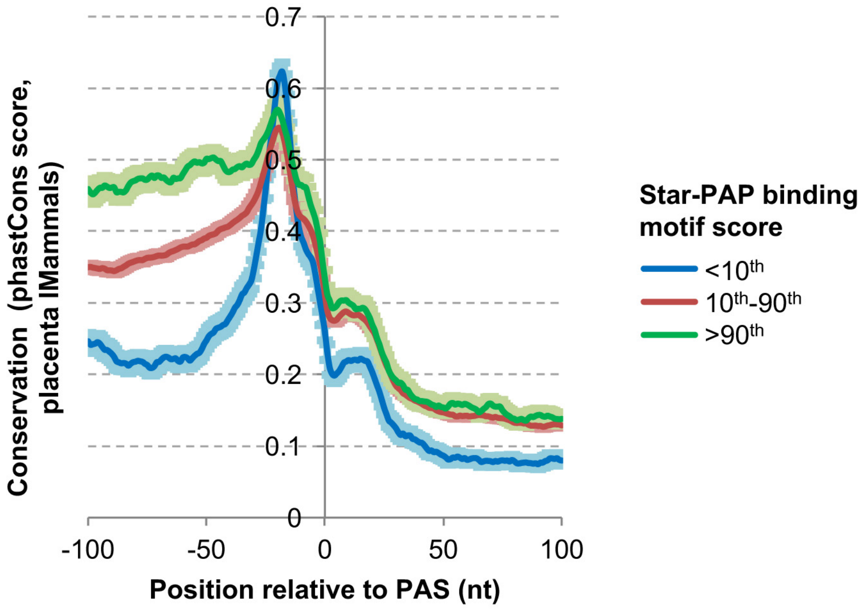

Figure S11. Conservation of PASs with different Star-PAP binding motif scores. Conservation is based on PhastCons score of placenta mammals downloaded from UCSC genome browser website. PAS +/- 100 nt region is shown. The Star-PAP binding prediction is based on the averaged motif score in the -100 to -41 nt region upstream of the PAS. The darker color line is the average of the scores. The boundary of the lighter color reflects the standard error of the mean (s.e.m).

Gene expression

APA regulation

siStar-PAP

	UP	DN	NC
Shortened	58	281	309
Lengthened	79	282	354
No change	118	419	522

Ratio DN/UP	*P*-value
4.8	0.126
3.6	
3.6	

siPAPα

	UP	DN	NC
Shortened	122	225	337
Lengthened	211	315	485
No change	173	272	420

Ratio DN/UP	*P*-value
1.8	0.155
1.5	
1.6	

siPAPγ

	UP	DN	NC
Shortened	108	195	354
Lengthened	169	178	407
No change	221	269	593

Ratio DN/UP	*P*-value
1.8	0.001
1.1	
1.2	

siPAPα+siPAPγ

	UP	DN	NC
Shortened	110	208	290
Lengthened	227	304	481
No change	170	264	387

Ratio DN/UP	*P*-value
1.9	0.020
1.3	
1.6	

siStar-PAP (rep2)

	UP	DN	NC
Shortened	37	54	174
Lengthened	43	70	210
No change	178	413	1351

Ratio DN/UP	*P*-value
1.5	0.776
1.6	
2.3	

Figure S12. Relationship between 3'UTR APA regulation and gene expression change. APA regulation was based on SAAP analysis (FDR=0.05). Gene expression was based on all PASS reads mapped to a gene. Regulation was based on Fisher's Exact test (P<0.01) and fold change>1.4. UP, upregulation; DN, downregulation; NC, no significant change. The ratio of DN/UP was shown. The *P*-value testing the association between APA and gene expression change was based on the red numbers in each table using the Fisher's Exact Test.

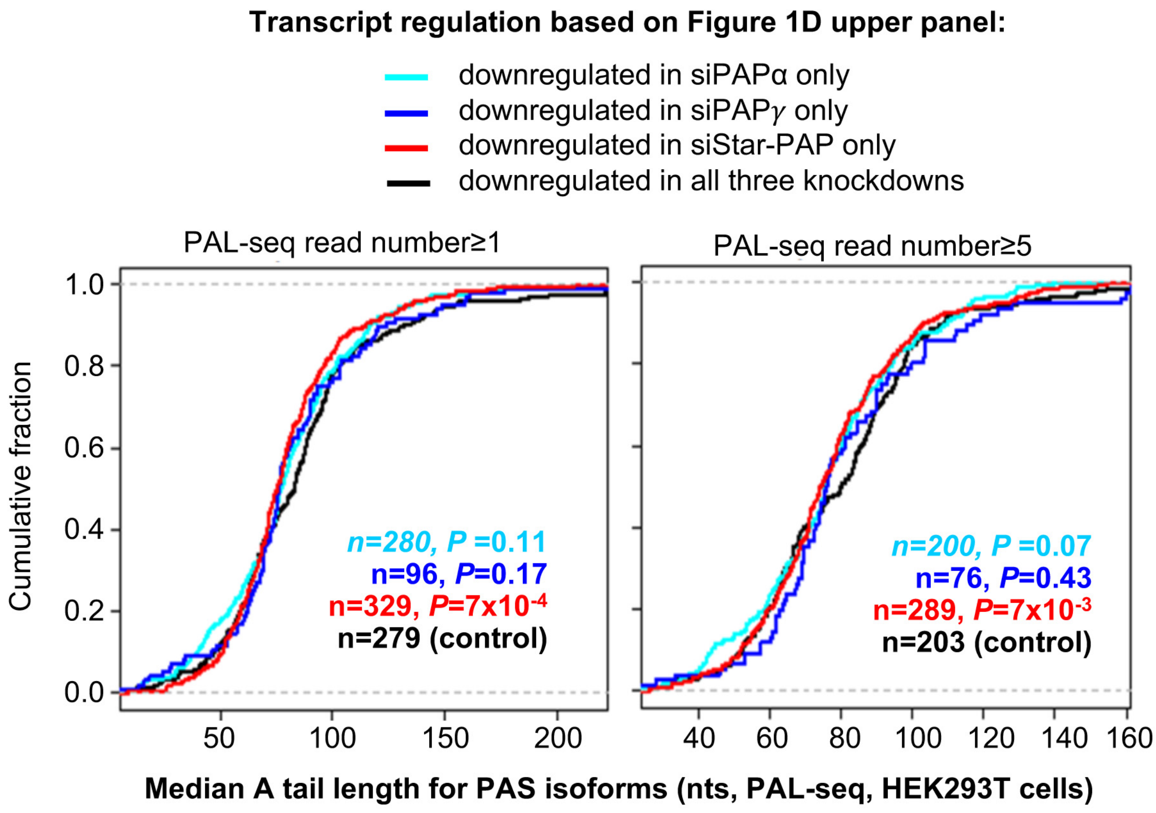

Figure S13: Poly(A) tail length for transcripts in different regulation groups after three PAP knockdowns (Based on Figure 1D upper panel). *P*-value was based on K-S test comparing transcripts downregulated in single PAP knockdown only vs. down-regulated in all three PAP knockdowns. Poly(A) tail length was based on the PAL-seq data for HEK293T cells (1) using the following method: The raw PAL-seq read fastq files were downloaded from SRA (SRP033369). The poly(A) tail normalized intensity were downloaded from GEO database (GSE52809). Read were mapped to hg19 genome using bowtie2. The reads uniquely mapped (MAPQ value >10) were used. The first nt of read was defined as poly(A) site. To determine the poly(A) tail length, a linear regression model was built using the A-length of synthetic spike-in RNAs and median value of normalized streptavidin fluorescence intensity. Then the poly(A) tail length was predicted by the model using the normalized streptavidin fluorescence intensity of the reads. The PAS of 3'READS data was matched with PAL-seq data by searching the +/- 24nt region of each PAS.

2. Supplementary Tables

Table S1: Mapping statistics of the RNA deep sequencing using 3'READS

	No. of raw reads	No. of uniquely mapped reads	No. of PASS reads
siCtrl	30,347,187	21,935,388	11,426,503
siPAPα	53,885,778	43,963,639	25,085,659
siPAPγ	30,511,200	24,294,691	14,794,886
siStar-PAP	39,128,685	30,491,659	16,549,350
siPAPα+siPAPγ	37,508,970	30,282,601	18,793,249
siCtrl (batch 2)	14,450,720	8,459,123	5,048,654
siStar-PAP (batch 2)	11,544,286	6,783,199	4,249,376

Table S2: Gene Ontology groups associated with down-regulated genes after KD

All KDs	siPAPα only	siPAPγ only	siStar-PAP only	Gene Ontology term
4.6	0.2	0.3	0.1	neuron differentiation
4.3	0.7	0.1	0.1	extracellular matrix organization
3.7	0.4	0.4	0	response to organic cyclic compound
3.6	0.2	0.1	0.2	glycerophospholipid metabolic process
3.5	0	0	0	regulation of Cdc42 protein signal transduction
0	2.7	0.8	0	post-embryonic development
0	2.5	0.4	0	dicarboxylic acid metabolic process
0.1	2.1	0.5	0	Golgi vesicle transport
1.2	0	2.3	0	cell-type specific apoptotic process
0.1	0	0	3.1	regulation of chromosome organization
0.5	0.3	0	2.5	organelle localization
0	0.1	0	2.5	chromosome organization
0	0	0.3	2.4	sister chromatid cohesion
0	0.1	0.3	2.2	alpha-amino acid catabolic process

Values are $-\log_{10}(P)$, where P was based on the Fisher's exact test. Genes with fold change >1.5 were selected for analysis. Only the Biological Process category of GO was analyzed. GO terms were sorted by significance in each group.

Table S3: Gene Ontology terms enriched for genes with APA changes

All KDs	siPAPα only	siPAPγ only	siStarPAP only	Gene Ontology term
8	0	0.5	0.6	mRNA metabolic process
4.7	0	0.2	0.9	RNA processing
3.7	0.3	0.7	0.1	protein folding
3.5	0.4	0.6	0	establishment of protein localization to membrane
3.5	0.1	0.6	0	cellular macromolecule catabolic process
0.2	2.4	0.4	0.2	negative regulation of type I interferon production
0.1	2	0	0.4	sterol metabolic process
0.3	0.2	3.5	0	regulation of translational initiation
0.9	0.3	2.2	0	establishment of protein localization to mitochondrion
0.8	0	2.1	0	protein import
0.2	0.6	2	0	response to ketone
0.4	0.4	2	0.5	negative regulation of multicellular organismal process

Values are $-\log_{10}(P)$, where P was based on the Fisher's exact test. Genes containing regulated APA sites (FDR = 0.05) were selected for analysis. Only the Biological Process category of GO was analyzed. GO terms were sorted by significance in each group.

3. Antibodies. Rabbit polyclonal anti-Star-PAP and anti-PIPKIα (Anderson's Lab homemade (2)); Goat polyclonal anti-EIF4A1 (Santa Cruz Biotechnology, #sc-14211); Rabbit monoclonal anti-PTEN (Cell Signaling Technology, #9559); Mouse monoclonal anti-Actin (MP Biomedical, #691002); Rabbit polyclonal anti-PKCδ (Santa Cruz Biotechnology, #sc-937); Rabbit polyclonal anti-PAPα (Bethyl Laboratories, #A301-010A); Rabbit polyclonal anti-PAPγ (Bethyl Laboratories, #A302-427A); Goat polyclonal anti-CKIα (Santa Cruz Biotechnology, #sc-6477); Rabbit polyclonal anti-CKIε (Bethyl Laboratories, #A302-135A).

4. Primers used for RT-PCR:

PTEN-fw: ACCAGGACCAGAGGAAACCT (CDS)

PTEN-rv: GCTAGCCTCTGGATTTGACG (CDS)

PTEN distal PAS-fw: AATACCAATATGATGTGTAC (RIP)

PTEN distal PAS-rv: CTTTAATCAGTTTTAAGTGGAG (RIP)

EIF4A1 distal PAS-fw: CTCCAGATCCCAGAGGCTCT (qRT-PCR and RIP)

EIF4A1 distal PAS-rv: CCTCCAGATTTGGTCCAGCA (qRT-PCR and RIP)

EIF4A1 reference-fw: GGCCGTAAAGGTGTGGCTAT

EIF4A1 reference-rv: AGGTCAGCAACATTGAGGGG

NEAT1 distal PAS-fw: GTAGTCTCGGGTATGCTG (qRT-PCR and RIP)

NEAT1 distal PAS-rv: TCTAATGAGTTTAGAACTCA (qRT-PCR and RIP)

NEAT1 reference-fw: GAGTCCTAGCATTGCAGGAG

NEAT1 reference-rv: GAATACAGGGCTTCCTGAGC

GPBP1 proximal PAS-fw: TCTCTCATACAGTTTGGGGTGA

GPBP1 proximal PAS-rv: ACCAACCCAAGTCCTCATTCC

GPBP1 reference-fw: TGTTGTCAGACCAGGGTTTTCA

GPBP1 reference-rv: GGACAGATCCAGTCAGTGTGA

CHAF1A distal PAS-fw: TTGCTGGCCTATTGGGGAAG

CHAF1A distal PAS-rv: CACTGCCTCGTTAGGTCCAG

CHAF1A reference-fw: GAGAGCAGGGGTGACGTATG

CHAF1A reference-rv: CGTGAAGCAGGACAAAGTGC

CYTH2 distal PAS-fw: TGCTTCCTTCAGCACCATGT

CYTH2 distal PAS-rv: GTGCACTCACTGAGGACCTC

CYTH2 reference-fw: CTGTGAGTGTGGACCCCTTC

CYTH2 reference-rv: CGGGGCAGCTCCGTAATAAA

GCLC 3’UTR-fw: ATGCCTGGTTTTCGTTTGCA

GCLC 3’UTR-rv: AGCTGTGGAACTCACACACACTCA

The GAPDH primers used for the real-time RT-PCR were the same as described previously (2).

5. siRNAs

siStar-PAP-1: AACUACGAGCTGCGAGAAA

siStar-PAP-2: GUGUGUUUGUCAGUGGCUU (This was picked for the series of experiments after the initial specificity characterization)

siPAPα-1: AAAAUCCCGACAAGGAAGAAU

siPAPα-2: GGAGACUGACUGCGUACUU (This was picked for the series of experiments after the initial specificity characterization)

siPAPγ-1: GCUGACAUUGAUGCACUUU

siPAPγ-2: GCUGGUGGUUCUUGGUAAA (This was picked for the series of experiments after the initial specificity characterization)

PTEN 3'UTR-specific siRNA-1: GGUAAAGUUAGAGACAACU (This was picked for the indicated experiments after the initial specificity characterization)

PTEN 3'UTR-specific siRNA-2: UAACACCUCACCAUUGAAA

Scrambled Control, PIPKIα, PKCδ, CKIα and CKIε siRNAs were used as described previously (2-5).

6. References

1. Subtelny, A.O., Eichhorn, S.W., Chen, G.R., Sive, H. and Bartel, D.P. (2014) Poly(A)-tail profiling reveals an embryonic switch in translational control. *Nature*, **508**, 66-71.

2. Mellman, D.L., Gonzales, M.L., Song, C., Barlow, C.A., Wang, P., Kendziorski, C. and Anderson, R.A. (2008) A PtdIns4,5P2-regulated nuclear poly(A) polymerase controls expression of select mRNAs. *Nature*, **451**, 1013-1017.

3. Gonzales, M.L., Mellman, D.L. and Anderson, R.A. (2008) CKIalpha is associated with and phosphorylates star-PAP and is also required for expression of select star-PAP target messenger RNAs. *The Journal of biological chemistry*, **283**, 12665-12673.

4. Laishram, R.S., Barlow, C.A. and Anderson, R.A. (2011) CKI isoforms alpha and epsilon regulate Star-PAP target messages by controlling Star-PAP poly(A) polymerase activity and phosphoinositide stimulation. *Nucleic Acids Res*, **39**, 7961-7973.

5. Li, W., Laishram, R.S., Ji, Z., Barlow, C.A., Tian, B. and Anderson, R.A. (2012) Star-PAP control of BIK expression and apoptosis is regulated by nuclear PIPKIalpha and PKCdelta signaling. *Mol Cell*, **45**, 25-37.
